# A systematic comparison of FOSL1, FOSL2 and BATF-mediated transcriptional regulation during early human Th17 differentiation

**DOI:** 10.1093/nar/gkac256

**Published:** 2022-05-03

**Authors:** Ankitha Shetty, Subhash Kumar Tripathi, Sini Junttila, Tanja Buchacher, Rahul Biradar, Santosh D Bhosale, Tapio Envall, Asta Laiho, Robert Moulder, Omid Rasool, Sanjeev Galande, Laura L Elo, Riitta Lahesmaa

**Affiliations:** Turku Bioscience Centre, University of Turku and Åbo Akademi University, Turku 20520, Finland; InFLAMES Research Flagship Center, University of Turku, Turku 20520, Finland; Centre of Excellence in Epigenetics, Department of Biology, Indian Institute of Science Education and Research (IISER), Pune 411008, India; Turku Bioscience Centre, University of Turku and Åbo Akademi University, Turku 20520, Finland; Center for Immunity and Immunotherapies, Seattle Children’s Research Institute, Seattle, WA 98101, USA; Turku Bioscience Centre, University of Turku and Åbo Akademi University, Turku 20520, Finland; InFLAMES Research Flagship Center, University of Turku, Turku 20520, Finland; Turku Bioscience Centre, University of Turku and Åbo Akademi University, Turku 20520, Finland; InFLAMES Research Flagship Center, University of Turku, Turku 20520, Finland; Turku Bioscience Centre, University of Turku and Åbo Akademi University, Turku 20520, Finland; InFLAMES Research Flagship Center, University of Turku, Turku 20520, Finland; Turku Bioscience Centre, University of Turku and Åbo Akademi University, Turku 20520, Finland; Department of Biochemistry and Molecular Biology, Protein Research Group, University of Southern Denmark, Campusvej 55, Odense M, DK 5230, Denmark; Turku Bioscience Centre, University of Turku and Åbo Akademi University, Turku 20520, Finland; Turku Bioscience Centre, University of Turku and Åbo Akademi University, Turku 20520, Finland; InFLAMES Research Flagship Center, University of Turku, Turku 20520, Finland; Turku Bioscience Centre, University of Turku and Åbo Akademi University, Turku 20520, Finland; InFLAMES Research Flagship Center, University of Turku, Turku 20520, Finland; Turku Bioscience Centre, University of Turku and Åbo Akademi University, Turku 20520, Finland; InFLAMES Research Flagship Center, University of Turku, Turku 20520, Finland; Centre of Excellence in Epigenetics, Department of Biology, Indian Institute of Science Education and Research (IISER), Pune 411008, India; Department of Life Sciences, Shiv Nadar University, Delhi-NCR; Turku Bioscience Centre, University of Turku and Åbo Akademi University, Turku 20520, Finland; InFLAMES Research Flagship Center, University of Turku, Turku 20520, Finland; Institute of Biomedicine, University of Turku, Turku, Finland; Turku Bioscience Centre, University of Turku and Åbo Akademi University, Turku 20520, Finland; InFLAMES Research Flagship Center, University of Turku, Turku 20520, Finland; Institute of Biomedicine, University of Turku, Turku, Finland

## Abstract

Th17 cells are essential for protection against extracellular pathogens, but their aberrant activity can cause autoimmunity. Molecular mechanisms that dictate Th17 cell-differentiation have been extensively studied using mouse models. However, species-specific differences underscore the need to validate these findings in human. Here, we characterized the human-specific roles of three AP-1 transcription factors, FOSL1, FOSL2 and BATF, during early stages of Th17 differentiation. Our results demonstrate that FOSL1 and FOSL2 co-repress Th17 fate-specification, whereas BATF promotes the Th17 lineage. Strikingly, FOSL1 was found to play different roles in human and mouse. Genome-wide binding analysis indicated that FOSL1, FOSL2 and BATF share occupancy over regulatory regions of genes involved in Th17 lineage commitment. These AP-1 factors also share their protein interacting partners, which suggests mechanisms for their functional interplay. Our study further reveals that the genomic binding sites of FOSL1, FOSL2 and BATF harbour hundreds of autoimmune disease-linked SNPs. We show that many of these SNPs alter the ability of these transcription factors to bind DNA. Our findings thus provide critical insights into AP-1-mediated regulation of human Th17-fate and associated pathologies.

## Graphical Abstract

**Graphical Abstract:**
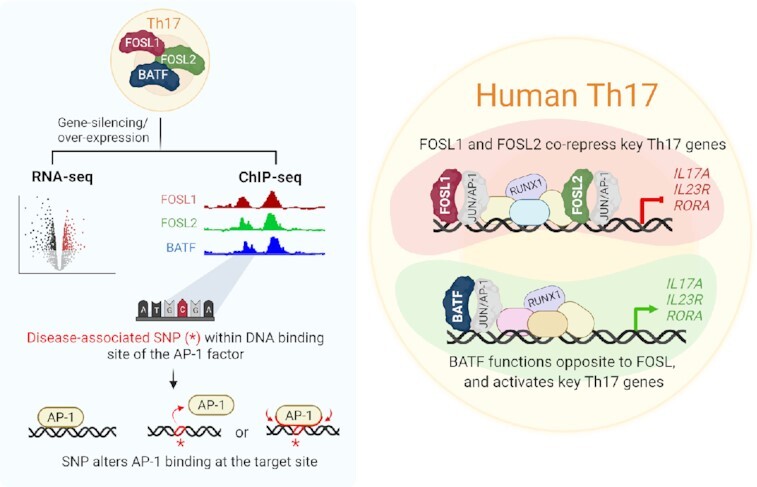
A systematic comparison of FOSL1, FOSL2 and BATF-mediated transcriptional regulation during early human Th17 differentiation.

## INTRODUCTION

Th17 cells play a central role in mucosal host defense against extracellular bacteria and fungi. Their uncontrolled response, however, can lead to autoimmune conditions such as rheumatoid arthritis (RA), multiple sclerosis (MS) and inflammatory bowel disease (IBD). Characterization of the molecular circuits that dictate Th17 cell-function is thus critical for developing therapeutic strategies against immune-mediated disorders. Primarily, Th17 cell-differentiation is initiated when naive CD4^+^ T-cells are exposed to IL-6 and TGF-β (with or without IL-1β or IL-23). The early stages of differentiation involve signaling cascades that endorse lineage-defining gene-expression programs and restrict the diversification to other T-helper (Th) cell fates. These events are dictated by a well-coordinated network of transcription factors (TFs), many of which have been functionally explored in both human and mouse. Studies using gene-knockout mouse models have demonstrated how pioneer factors, such as STAT3, BATF and IRF4, nucleate key Th17-defining proteins (RORγT, RORα) (1) over cytokine gene loci (*IL-17A, IL17F* and *IL22)* (2,3).

The AP-1 complex, which is constituted by JUN, FOS and ATF family proteins, plays a crucial role in transcriptional regulation of Th cell differentiation (4–7). Previous studies from our lab have revealed that the FOS family proteins, FOSL1 and FOSL2 (also known as *FRA-1* and *FRA-2*), are differentially expressed during initial stages of human Th17 polarization (8,9). Collectively termed as FOS-like (FOSL) proteins, these are paralogous transcription factors that have limited sequence similarity and can perform different functions. Their importance in the regulation of embryonic development, cancer progression, ECM synthesis and immune cell responses is well-established (7,10–15). In addition, these factors exert opposing effects on murine Th17-development. While *FOSL1* positively regulates the process in mouse, *FOSL2* acts a repressor of the lineage (2,16). Nonetheless, the Th17-regulatory roles of these factors have not been confirmed in human. The heterogeneity between human and mouse with regard to cytokine-responses, T cell-activation, γ/δ T-cell function and interferon signaling, is quite well-known (17). Significant differences have also been reported for the proteomic profiles of early-differentiating Th17 cells in the two species (9). Moreover, genes such as *AHR* (18–20), *PRDM1* (21,22) and *SATB1* (9), have been found to exhibit divergent effects on human and mouse Th17 function. These discrepancies should be especially borne in mind while extrapolating information from murine studies for therapeutic interventions. They further underscore the need to validate murine gene-functions using human cells. The inadequacy of such corroborative studies in the field, could hinder disease-research.

FOS and ATF proteins lack a transactivation domain, and thus need to heterodimerize with JUN and other factors to execute their gene-regulatory roles (7,23,24). Since the resulting transcriptional activity is dictated by both of the proteins forming the dimer, dissecting the individual function of the monomers has proved to be challenging. Findings across cell types have identified both cooperative and antagonistic relationships among AP-1 proteins, in a context-specific manner (7,24). Thus, investigating the AP-1 complex requires more comprehensive approaches where the molecular interplay between its members could be addressed. Such interrelated functions are previously reported for FOSL2 and BATF in murine Th17 cells, where the two factors bind over lineage-specific gene loci and regulate Th17-fate in an opposite fashion. BATF promotes murine Th17-differentiation, whereas FOSL2 inhibits the process (2,25). Importantly, the functional relationship between FOSL2 and BATF is poorly explored in human T-cells and the link between FOSL1 and BATF stands undetermined in either of the species.

In the present study, we investigated the individual and related roles of FOSL1, FOSL2 and BATF in regulating human Th17 cell-identity, while highlighting species-specific differences. By combining global gene-expression analysis and genome-wide occupancy studies, we dissected the genes that are directly regulated by these TFs. Our results demonstrate an evident coordination between FOSL1 and FOSL2 functions, while verifying their antagonistic relationship with BATF in human Th17 cells. Further analysis revealed that the genomic regions bound by these AP-1 proteins harbour hundreds of disease-linked single nucleotide polymorphisms (SNPs), many of which altered the ability of these proteins to bind DNA. Disrupting the binding-affinities of these TFs to their target gene-regulatory sites could subsequently alter their roles in Th17-regulation, and thereby contribute to disease development.

## MATERIALS AND METHODS

### Primary human CD4^+^ T-cell isolation and Th17 polarization (differentiation)

Human cord blood mononuclear cells (CBMCs) were isolated from the umbilical cord blood of healthy neonates (Turku University Central Hospital, Turku, Finland) using the Ficoll- Paque density gradient centrifugation (Ficoll-Paque PLUS; GE Healthcare). Naive CD4^+^ T-cells were further purified using CD4^+^ Dynal positive selection beads (Dynal CD4 Positive Isolation Kit; Invitrogen). CD4^+^ T-cells were stimulated with plate-bound α-CD3 (3.75 μg/ml; Beckman Coulter, Cat. no. IM1304) and soluble α-CD28 (1 μg/ml; Beckman Coulter, Cat. no. IM1376) in X-vivo 20 serum-free medium (Lonza). X-vivo 20 medium was supplemented with L-glutamine (2 mM, Sigma-Aldrich) and antibiotics (50 U/ml penicillin plus 50 μg/ml streptomycin; Sigma-Aldrich). Th17 polarization was induced using a cytokine cocktail of IL-6 (20 ng/ml; Roche), IL-1β (10 ng/ml) and TGF-β (10 ng/ml), in the presence of neutralizing anti-IFN-γ (1 μg/ml) and anti-IL-4 (1 μg/ml) to block Th1 and Th2 differentiation, respectively. For the differentiation control, activated T-cells (Th0) were used, which were obtained by stimulating naive CD4^+^ T-cells with α-CD3 and α-CD28 in the presence of neutralizing antibodies, but without the polarizing cytokines. All cytokines and neutralizing antibodies used in the study were purchased from R&D Systems unless otherwise stated. All cultures were maintained at 37°C in a humidified atmosphere of 5% (v/v) CO_2_/air.

### Expression analysis of FOSL1 and FOSL2 during human Th17 cell differentiation

FOSL transcript levels were analysed using transcriptome data from our previously published study (8). Human umbilical cord blood-derived naive CD4^+^ T-cells were cultured for Th0 and Th17 conditions as described above. Three biological replicates of samples were collected at 0, 0.5, 1, 2, 4, 6, 12, 24, 48 and 72 h time points. RNA was isolated (RNeasy Mini Kit, QIAGEN) and DNase treated (RNase-Free Dnase Set; QIAGEN). RNA-seq with 50 nt read length was performed at Illumina sequencing service provider with HiSeq 2000 instrument. Bioconductor package edgeR was used to define differential expression between Th17 and Th0 conditions. Further details on cell culture, RNA-sequencing and differential gene expression analysis are provided in the methods section of the original article (doi: 10.18632/oncotarget.7963).

### RNAi silencing

CD4^+^ T-cells from umbilical cord blood were suspended in Opti-MEM I (Invitrogen) and transfected with the respective gene-targeting siRNA using the Lonza nucleofection technique with the U-14 Program on the Amaxa Nucleofector™ device. Control cells were treated with the same amount of non-targeting or Scramble siRNA (SCR) (Sigma).

#### STAT3 and BATF knockdown (KD)

Four million cells were transfected with 6 μg of STAT3- or BATF-targeting siRNA, after which the cells were rested at 37°C for 36–40 h in RPMI 1640 medium (Sigma-Aldrich) supplemented with pen/strep, l-glutamine (2 mM) and 10% FCS. Cells were subsequently activated and cultured under Th17 conditions. For identification of global targets by RNA-seq, SCR and BATF KD Th17 cells were harvested at 24 h and 72 h of differentiation. All siRNA sequences are provided in Supplementary Table S1. A pool of two siRNAs was used for silencing BATF.

#### FOSL knockdown (KD) and double KD (DKD)

For the single KD experiments, four million cells were transfected with either 5 μg of FOSL1 or FOSL2-targeting siRNAs or 5μg of Scramble control siRNA. The rest of the protocol is as described above. For the double KD (DKD) experiments, four million cells were nucleofected with 10 μg of FOSL-targeting siRNA (i.e. 5 μg FOSL1 + 5 μg FOSL2 siRNA) or 10 μg of Scramble siRNA. Single KD controls (KD) for FOSL1/FOSL2 were also maintained (i.e. 5 μg FOSL1 or FOSL2 siRNA + 5 μg scramble control siRNA). For identification of global targets by RNA-seq : SCR, single KD and DKD Th17 cells were harvested at 24 h and 72 h of differentiation. All siRNA sequences are provided in Supplementary Table S1.

### FOSL over-expression (OE) and double OE

#### Generating in-vitro transcribed (IVT) RNA

To generate linearized vectors for the IVT reaction, the T7 promoter containing plasmids: empty pGEM-GFP64A, pCMV6-AC-GFP-FOSL1 (Origene, Cat. no. RG202104) and pCMV6-AC-GFP-FOSL2 (Origene, Cat. no. RG204146), were *in vitro* digested using the restriction enzymes Spe1 (NEB, Cat. no. R0133), Xma1 (NEB, Cat. no. R0180) and Ssp1 (NEB, Cat. no. R3132), respectively. Digestion was performed for 1 h using Cut Smart Buffer (NEB, Cat. no. B7204S). Next, using the generated templates, IVT RNA was produced using Cell Script MessageMAXTM T7 ARCA-Capped Message Transcription Kit (Cell Script, Cat. no. C-MMA60710), by following manufacturer's instructions. 10 M lithium chloride (LiCl) was used to precipitate the product (−20°C, O/N), followed by 70% ethanol washes (two washes, each followed by a 10-min centrifuge spin) and resuspension in nuclease-free water. The size of the RNA was confirmed using BioRad Experion or Agilent Bioanalyzer at this step. The RNA was further poly-adenylated using Cell script A-Plus™ Poly(A) Polymerase Tailing Kit (Cell Script, Cat. no. C-PAP5104H). LiCl precipitation was repeated, and the final pellet was resuspended in nuclease-free water. RNA concentration was determined using a Nanodrop™ detector (Thermo Scientific) and the IVT RNA was stored at −80°C till further use.

#### Nucleofection

For the double over-expression (DOE) experiments, 4 million cells were nucleofected with FOSL1 + FOSL2 IVT RNA (i.e. 56 pmol FOSL1-GFP + 56 pmol FOSL2-GFP RNA) or control GFP RNA (112 pmol). Single over-expression controls (OE) for FOSL1/FOSL2 were also maintained (i.e. 56 pmol FOSL1 or FOSL2-GFP RNA + 56 pmol control GFP RNA). We ensured equimolar RNA amounts across the different nucleofection conditions. Cells were rested for 18–20 h post-nucleofection and further cultured under Th17 conditions. For identification of global targets by RNA-seq: GFP, single OE and DOE Th17 cells were harvested at 72 h of differentiation.

### Gene-expression analysis

#### RNA Isolation and RNA-Seq Sample Preparation

RNA was isolated (RNeasy Mini Kit; QIAGEN, Cat. no. 74104) and given on-column DNase treatment (RNase-Free DNase Set; QIAGEN) for 15 min. The removal of genomic DNA was ascertained by an additional treatment of the samples with DNase I (Invitrogen, Cat. no. 18068-015). After RNA quantification (using NanoDrop™ 2000) and quality control (using BioRad Experion or Agilent Bioanalyzer), libraries for RNA-Seq were prepared for three biological replicates. The high quality of the libraries was confirmed with Advanced Analytical Fragment Analyzer (Advanced Analytical Technologies, Heidelberg, Germany) or with Agilent Bioanalyzer, and the concentrations of the libraries were quantified with Qubit^®^ Fluorometric Quantitation (Life Technologies, ThermoFisher). Sequencing was performed at the Finnish Functional Genomics Centre (FFGC) using HiSeq3000 Next-Generation Sequencing platform.

#### Alignment and Differential Expression Analysis

50-bp single-end reversely-stranded sequencing reads were checked for quality using FastQC (v.0.11.14) and MultiQC (v.1.5) (26). High-quality reads were aligned to the human reference genome (hg38) using R (v.3.6.1)/Bioconductor(v.3.9) (27) package Rsubread (v.1.34. (28) which was also used for producing the gene-wise read counts based on RefSeq gene annotations. Statistical testing and differential expression analysis was performed using Bioconductor package ROTS (v.1.12.0) (29). For each comparison, the expressed genes (CPM expression value > 1) in at least 50% of the replicates in one of the compared sample groups were included in the statistical testing. DE genes were identified with cut-offs of false discovery rate (FDR) ≤ 0.1 and absolute fold-change (specified in the corresponding figure legends and results section). The DE gene heatmaps were produced using R package pheatmap (v. 1.0.12).

### ChIP-seq analysis

#### Sample preparation

CD4^+^ T-cells were cultured under Th17 cell polarizing conditions for 72 h. Chromatin was prepared from 40–50 million cells using Diagenode Chromatin shearing optimization kit (Cat. no. C01010055) and further subjected to sonication using Bioruptor sonicator (Diagenode) to obtain chromatin fragments of 100–500 bp. Fragmented chromatin was diluted using chromatin dilution buffer (1 mM EDTA (pH 8), 0.5 mM EGTA (pH 8), 10 mM Tris (pH 8), 200 mM NaCl, 0.1% Na-Deoxycholate, 0.5% *N*-lauroylsarcosine). 300 μl of Triton-X buffer (1% Triton X-100, 0.1% sodium deoxycholate in Tris–EDTA buffer (pH 8)) was added to every 1ml of diluted chromatin.

10–12 μg of FOSL1 (Santacruz Biotechnology, Cat no. sc-28310), FOSL2 (Cell Signaling Tech, Cat. no. 19967) or BATF (Cell Signaling, Cat. no. 8638) antibody was pre-incubated with magnetic beads for crosslinking (Dynal Biotech/Invitrogen, Cat. no. 112.04) in blocking buffer (5 mg/ml BSA in PBS). The fragmented chromatin was then incubated overnight (4°C, on rotate) with the Ab-bead complex. This was followed by subsequent washing steps with RIPA buffer (50 mM HEPES–KOH (pH 7.6), 500 mM LiCl, 1mM EDTA, 1% NP-40, 0.7% Na-deoxycholate). Final wash was given with Tris-EDTA buffer (10 mM Tris–Cl (pH 8), 1 mM EDTA (pH 8)). Chromatin was finally eluted at 65°C using Elution buffer (10 mM Tris (pH 8), 1mM EDTA, 1% SDS) (30 min, mixer conditions). The crosslinks were further reversed (65°C for 12–16 h, mixer conditions), treated with proteinase K (Ambion Inc., Cat. no. AM2546) and RNase A (Thermo Scientific, Cat. no. ENO531) and then purified using QIAquick PCR purification kit (QIAGEN, Cat. no. 28104). DNA libraries were prepared using two biological replicates of each TF ChIP and sequenced on HiSeq4000 or Miseq (Fasteris Life Sciences, Plan-les-Ouates, Switzerland).

#### Analysis

75-bp paired-end reads were obtained, and quality control was performed with FastQC (v. 0.11.4) (https://www.bioinformatics.babraham.ac.uk/projects/fastqc/). The adapter sequences present in the raw reads were trimmed using TrimGalore! (v. 0.4.5) (https://www.bioinformatics.babraham.ac.uk/projects/fastqc/), and the trimmed reads were mapped to the hg38 reference genome using Bowtie2 (v. 2.3.3.1) (30). Duplicate reads were marked with Picard tools (v. 2.20.2) (https://broadinstitute.github.io/picard/) MarkDuplicates function and reads with mapping quality <30 were filtered out using samtools (v. 1.9) (31). Sample quality was controlled by calculating cross-correlation scores and the non-redundant fraction with phantompeakqualtools (v. 1.2) (32,33) and preseq (v. 2.0) (34), respectively. Peaks were called using MACS2 (v. 2.1.0) (35), and reproducible peaks were identified using IDR (36) with an FDR cut-off of 0.01. R packages ChIPpeakAnno (v. 3.21.7) (37) and EnsDb.Hsapiens.v86 (v. 2.99.0) were used to annotate the peaks and identify peaks common to all three transcription factors with a minimum overlap of 200 bp. In addition to the nearest features, the annotation includes any features that overlap the peaks resulting in more than one row per peak for many of the peaks in the excel files provided as supplementary files. The number of peaks common to the transcription factors reported in the main text and in the Venn diagrams, is the minimum number of overlapping peaks. Enriched transcription factor binding site motifs within the peaks were identified by Homer (v. 4.11) using both *de novo* and known motifs. A 200-bp region size was used for motif finding.

#### Re-alignment of publicly available H3K27Ac dataset

We used H3K27Ac ChIP-seq data from a published study (38). Data was acquired from GEO (GSE101389) for the activated human Th17 subset (Day 5 Th17 + IL-10-). For further details, refer to the original publication (doi: 10.1038/s41590-018-0200–5). Since the original alignment of the data was to hg19, raw reads were obtained and re-aligned to hg38 with Burrows-Wheeler alignment (BWA). Bigwig files were generated using bam coverage, normalized to Rpkm. Input subtracted files were generated using Compare Utility from deepTools.

### SNP analysis

SNPs associated with 11 auto-immune diseases were analysed for enrichment within the TF ChIP peaks using the R package snpEnrichR (v. 0.0.1) (39). The SNPs were queried from the NHGRI-EBI GWAS catalogue; SNPs from studies with meta-analysis of more than one disease and from populations other than Caucasian were excluded from further analysis, and correlated SNPs were clumped (distance = 1000 kb, LD *r*^2^ = 0.8). Random SNP sets matching the disease-associated SNPs were produced using SNPsnap (40) server with default parameters except distance = 1000 kb, LD buddies ± 20%, *r*^2^ = 0.8. Proxy SNPs for both disease-associated and random SNPs were calculated using Plink (v1.90b6.16) (www.cog-genomics.org/plink/1.9/) (41) from 1000 genomes EUR population. SNPs and their proxies (distance within 100 kb and *r*^2^ > 0.8, determined from 1000 genomes Eur population) overlapping the peaks, were identified and annotated to the nearest neighbour gene using ChIPpeakAnno. SNPs and proxies overlapping known transcription factor motifs were identified using annotatePeaks.pl from Homer. Motifs were searched within a 30-bp region around each SNP coordinate.

### DNA affinity precipitation assay (also known as DAPA)

DNA affinity precipitation assay experiments were performed as described in (42–44) with minor modifications. In brief, annealed biotinylated sense and non-biotinylated antisense bait oligonucleotides were purchased from Integrated DNA Technologies, Inc. Oligonucleotide probes containing the FOSL1, FOSL2 or BATF DNA binding motifs were designed with or without the SNP mutation (Supplementary Table S9). Mutations introduced to the oligonucleotides are highlighted in red (Supplementary Table S9). BATF-specific and mutated sequences were used as a positive control. Neutravidin beads (Ultralink immobilized neutravidin protein, Pierce) were washed 4× with buffer A (10 mM HEPES pH 7.9, 60 mM KCl, 2 mM EDTA, 1 mM EGTA, 0.1% Triton X-100, 1 mM DTT, and protease and phosphatase inhibitors from Roche). Annealed oligonucleotides were incubated with 25 μl of beads in 200 μl buffer A for 1.5 h at 4°C with rotation at 360° rotator, followed by 4× washes with buffer A. Nuclear fraction isolated from Th17 cells cultured for 72 h (Nuclear and Cytoplasmic Extraction Reagents kit, Pierce) was subjected to buffer 2 (10 mM HEPES, pH 7.9, 2 mM EDTA, 1 mM EGTA, 0.1% Triton X-100, 1 mM DTT, and protease and phosphatase inhibitors from Roche) to dilute any KCl salt. Pre-clearing was performed with unconjugated beads by incubating for 1.5 h in a 360° rotator at 4°C. Binding reactions of pre-cleared nuclear fraction with bead-conjugated oligonucleotides was performed for 4 h at 4°C, followed by washing four times with buffer A. Protein pull-down precipitates were eluted by incubating beads at 95°C for 5 min in 50 μl of 2× SDS buffer (125 mM Tris–HCl, pH 6.8, 4% w/v SDS, 20% glycerol, 100 mM DTT). FOSL1, FOSL2 and BATF protein was analysed by western blotting using rabbit monoclonal FOSL1 antibody (D80B4), rabbit monoclonal FOSL2 antibody (D2F1E) and rabbit monoclonal BATF antibody (D7C5) from Cell Signaling Technology.

### Graphical representation, Venn diagrams and Statistical analysis

All graphs were plotted using GraphPad Prism software (V8.3.0). Two-tailed Student's *t*-test was used to calculate statistical significance. Venn diagrams were generated using Biovenn (45) or Venny (https://bioinfogp.cnb.csic.es/tools/venny/index.html). Workflow illustrations for the study were prepared used BioRender.com.

## RESULTS

### FOSL1 and FOSL2 are upregulated during induction of human Th17-fate

**Figure 1.**
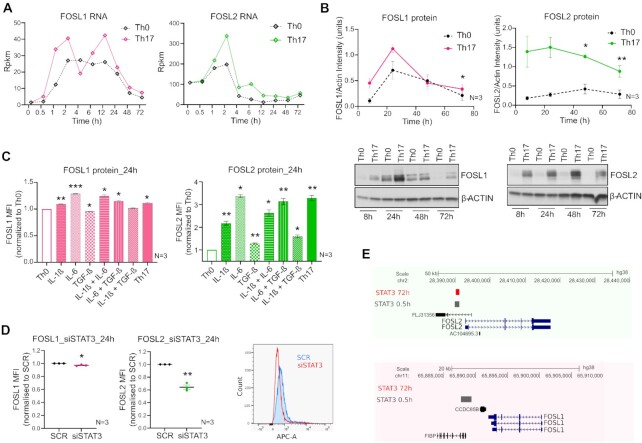
Expression of FOS-like proteins during human Th17 differentiation. (**A**) Rpkm values are plotted for FOSL1 (left) and FOSL2 (right) RNA at different time points of activation (Th0) or Th17-differentiation, using our published RNA-seq data (8). (**B**) Immunoblot images (lower panel) show FOSL1 (left) and FOSL2 (right) protein levels in Th0 and Th17-polarizing cells, over a time-course. Actin was used as loading control. Blots from three biological replicates were quantified using ImageJ and FOSL intensity values (normalized to actin) were plotted as a line graph in the above panel. (**C**) Flow cytometry analysis of FOSL1 (left) and FOSL2 (right) expression in naive CD4^+^ T-cells cultured for 24 h, under conditions of activation (Th0), Th17-differentiation, or activation in presence of the Th17-cytokines (used either alone or in combination). Bar plot shows median fluorescence intensity (MFI) values normalized to Th0, for three biological replicates. Statistical significance was calculated by comparing each condition to Th0. (**D**) Flow cytometry analysis of FOSL1 (left) and FOSL2 (right) protein levels in non-targeting (SCR) versus STAT3 KD cells, at 24 h of Th17 polarization. Graph shows MFI values normalized to SCR for three biological replicates. Adjoining histogram (flow cytometry analysis) confirms the depletion of STAT3 protein levels in the KD cells at 24 h of Th17 polarization. All graphs in panels B–D show mean ± standard error of the mean (SEM). Statistical significance is calculated using two-tailed Student's t test (*p < 0.05; **p < 0.01, ***p < 0.001). (**E**) UCSC genome browser snapshots indicate the binding of STAT3 over the promoter of FOSL2 (above panel) and not FOSL1 (below panel), in Th17 cells cultured for 0.5 and 72 h. Figures were derived using bed files of STAT3 ChIP-seq data from our published study (43).

To study the role of FOSL1 and FOSL2, we first analysed their expression profile during human Th17 differentiation. To this end, we used RNA-seq data from our previously published study (8) wherein we investigated the global gene expression profile of human Th17 cells over a time course. Here, naive CD4^+^ T-cells (T-helper precursor or Thp) derived from human umbilical cord blood were stimulated with anti-CD3 and anti-CD28 antibodies in the presence of polarizing cytokines (IL-6, TGF-β and IL-1β), in order to induce Th17 cell-differentiation. Cells activated with anti-CD3 and anti-CD28 alone, were used as controls (TCR-activated cells known as Th0). We plotted FOSL expression data at different time points and realized that their levels were quite low in naive T-cells, but they were significantly upregulated upon activation or differentiation (Figure 1A). Interestingly, Th17 cells showed higher levels of FOSL1 and FOSL2 than Th0, at most time points. These changes were subsequently validated at the protein level, by immunoblot analysis (Figure 1B; Supplementary Figure S1A, B). While the differential upregulation of both proteins was the maximum at 24 h, FOSL2 showed a more prominent increase at all of the evaluated time points.

TCR signaling is already known to upregulate AP-1 activity (7,46). We thus wanted to investigate which of the Th17-polarizing cytokines increase FOSL expression above the TCR-induced levels (Th0). To achieve this, naive CD4^+^ T-cells were activated in the presence of different Th17 cytokines (IL-6, TGF-β or IL-1β), used either individually or in combination. FOSL1 and FOSL2 protein levels were then analysed at 24 h of differentiation, using flow cytometry (Figure 1C). Our results found IL-1β and IL-6 to significantly promote the expression of both proteins, as compared to Th0. IL-6 particularly showed a stronger effect by inducing a 2.3-fold increase in FOSL2, and a 0.3-fold increase in FOSL1 expression. We additionally found TGF-β to upregulate FOSL2 levels (relative to Th0), which complies with previous findings in cancer cells (47).

The IL-6/STAT3 signaling axis is known to drive the expression of FOS-like proteins (16,43,48,49). Given the importance of STAT3 in establishing Th17-cell identity (43,50), we wanted to determine if the IL-6-induced increase in FOSL expression requires STAT3 function in human Th17 cells. To address this, FOSL1 and FOSL2 levels were examined in STAT3-depleted Th17 cells, using flow cytometry analysis (Figure 1D). Loss of STAT3 significantly reduced FOSL2 expression, while it had a minimal effect on FOSL1. Notably, in an earlier study (43), we have found STAT3 to occupy the promoter region of *FOSL2* but not *FOSL1*, which might explain the more robust effect on the former (Figure 1E).

### IL-17 expression is co-inhibited by FOSL1 and FOSL2

**Figure 2.**
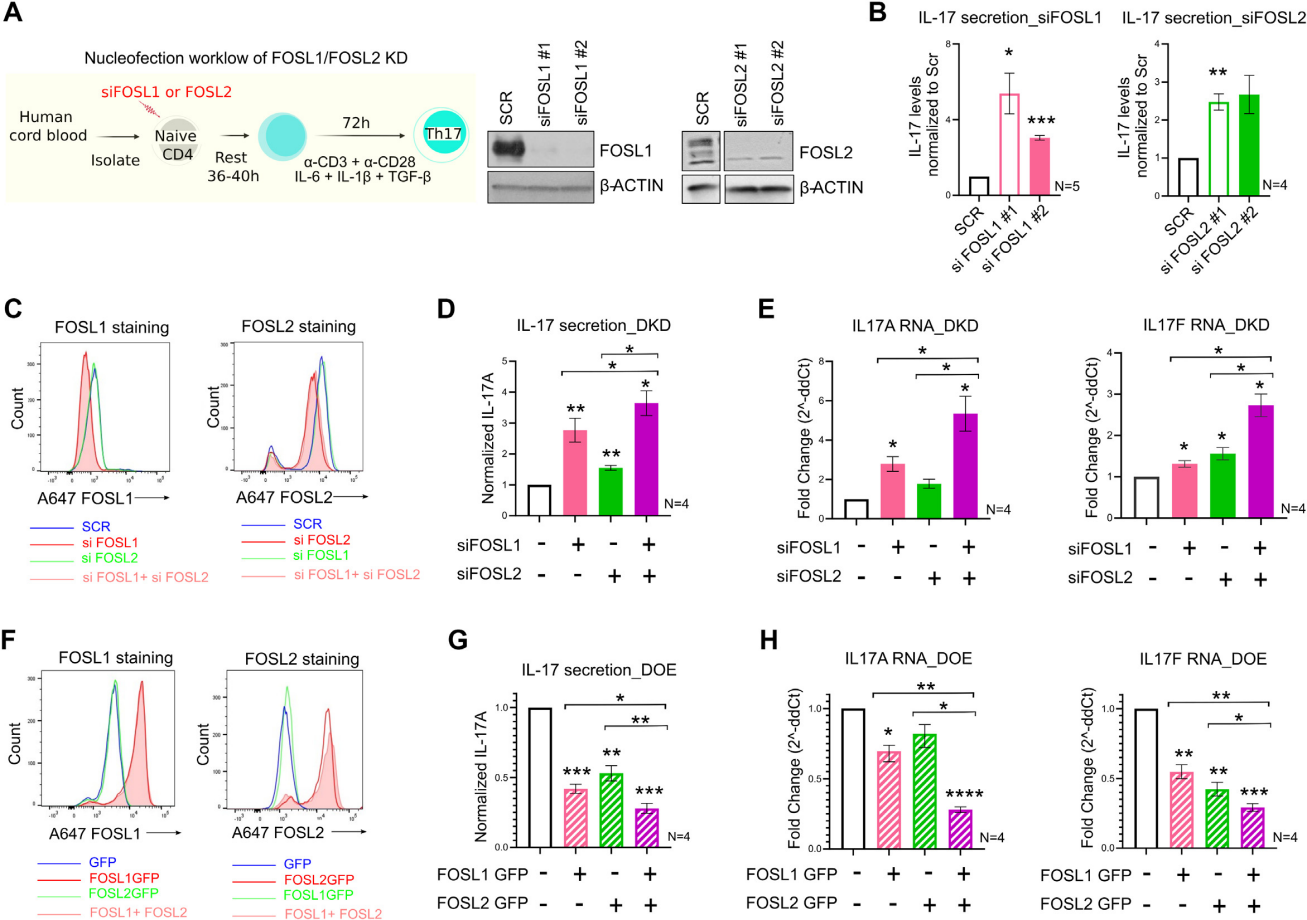
FOSL1 and FOSL2 negatively regulate IL-17 expression. (**A**) Nucleofection workflow for FOSL1/FOSL2 knockdown (KD) is shown in the left panel. Naive CD4^+^ T-cells were treated with two different siRNAs targeting FOSL1 or FOSL2. Cells were rested at 37°C in RPMI-medium, and further cultured under Th17-polarizing conditions. At 24 h post differentiation, knockdown was analysed using immunoblotting (right panel). Representative blots for three biological replicates are shown. (**B**) ELISA was used to estimate IL-17A secretion in supernatants of FOSL1 (left) and FOSL2-silenced (right) Th17 cells, at 72 h of polarization. Values were first normalized for cell count (live), and then normalized to SCR control. Data represents four or five biological replicates, as indicated. (**C**) Naive CD4^+^ T-cells were silenced for FOSL1, FOSL2 or both factors in parallel (double KD; DKD) and cultured under Th17-polarizing conditions for 24 h. Total FOSL1 (left) or FOSL2 (right) protein was stained (Alexa-647) and analysed by flow cytometry. Representative histograms for four biological replicates are shown. (**D**) Bar plot shows ELISA results for secreted IL-17A levels in supernatants of FOSL KD/DKD Th17 cells, at 72 h of polarization. Values were first normalized for cell count (live), and then normalized to SCR control. Data represents four biological replicates. (**E**) qRT-PCR analysis for measurement of IL-17A (left) and IL-17F (right) RNA levels in FOSL KD/DKD Th17 cells (72 h). Fold-change normalized to the control was plotted for four biological replicates. (**F**) Naive CD4^+^ T-cells were treated with *in-vitro* transcribed GFP-FOSL1 RNA, GFP-FOSL2 RNA or both (double OE; DOE). GFP RNA was used as nucleofection control. After resting the cells for 18–20 h, total FOSL1 (left) or FOSL2 (right) protein was stained (Alexa-647) and analysed by flow cytometry. Representative histograms for four biological replicates are shown. (**G, H**) Graphs show IL-17 secretion (panel G) and IL-17A/F RNA expression (panel H) in FOSL OE/DOE Th17 cells at 72 h of polarization, as assessed by ELISA and qRT-PCR analyses, respectively. ELISA values were first normalized for cell count (live), and then normalized to GFP control. Panel H depicts fold change normalized to control. Data represents four biological replicates. Plots in figures B, D, E, G, H show mean ± SEM. Statistical significance is calculated using two-tailed Student's *t* test (*p< 0.05, **p < 0.01, ***p < 0.001, ****p< 0.0001).

The early and sustained expression of FOS-like proteins suggests their potential involvement in steering Th17-differentiation. To determine their precise roles, we silenced each of these proteins individually with RNAi and probed for an effect on IL-17 cytokine, which is a key marker of the Th17 lineage. To ensure reproducibility and minimal off-target phenotypes, FOSL1 and FOSL2 were each targeted using two different siRNAs. Naive CD4^+^ T-cells were nucleofected and cultured according to the workflow in Figure 2A. The siRNA-efficacy was confirmed with western blotting (Figure 2A; Supplementary Figure S2A). Interestingly, depletion of FOSL1 or FOSL2 significantly increased IL-17 secretion at 72 h of polarization, which indicates a negative influence of these factors on human Th17 differentiation (Figure 2B). This further suggests that although FOSL2 function is similar in human and mouse (2), FOSL1 exhibits divergent roles in Th17-regulation of the two species (16).

Since FOSL1 and FOSL2 regulated IL-17 secretion in a similar fashion, we next examined if their simultaneous perturbation causes enhanced changes in Th17 effector functions. To this end, both siRNA knockdown (KD) and RNA-based over-expression (OE) strategies were used. For simultaneous silencing (double KD or DKD) of these factors, naive CD4^+^ T-cells were nucleofected with a combination of FOSL1- and FOSL2-targeting siRNAs, and flow cytometry was performed to confirm the parallel reduction in their protein levels (Figure 2C; Supplementary Figure S2B). Cells individually silenced for either of these factors served as single KD controls. In agreement with earlier findings (51,52), silencing FOSL1 did not alter FOSL2 expression, and vice-versa (Figure 2C). To further assess the effect of the KD on the expression of IL-17 cytokine, ELISA and qPCR analyses were performed at 72 h of polarization. Notably, co-depletion of FOSL1 and FOSL2 additively augmented IL-17 protein and RNA levels, relative to the single KD controls (Figure 2D, E).

To authenticate these findings, we simultaneously over-expressed the two proteins, using *in-vitro* transcribed (IVT) RNA. Naive CD4^+^ T-cells were nucleofected with a combination of FOSL1 and FOSL2 IVT RNAs (double OE or DOE), and flow cytometry analysis confirmed the lateral increase in their expression levels (Figure 2F; Supplementary Figure S2C). Parallel overexpression of the two proteins caused higher inhibition of IL-17 protein (ELISA analysis, Figure 2G) and RNA (qRT-PCR analysis, Figure 2H), as compared to the single OE controls. This strengthened our RNAi findings and confirmed the functional coordination between FOSL1 and FOSL2.

### Perturbing FOS-like proteins triggers important changes in Th17 gene expression program

To globally unravel the individual and combined gene targets of FOSL proteins, RNA-sequencing and differential expression (DE) analysis was performed for KD and DKD Th17 cells. For FOSL1 KD, FOSL2 KD and DKD conditions, respectively, our analysis detected 466, 1529 and 2000 DE genes at 24 h and 315, 150 and 1500 DE genes at 72 h of polarization (false discovery rate (FDR) ≤ 0.1) (Supplementary Table S2). A similar analysis was performed for OE and DOE Th17 cells (at 72 h) resulting in the identification of 30, 352 and 521 DE transcripts for FOSL1 OE, FOSL2 OE and DOE conditions, respectively (FDR ≤ 0.1) (Supplementary Table S3). It was thus evident that co-perturbation of these factors altered a higher number of genes.

**Figure 3.**
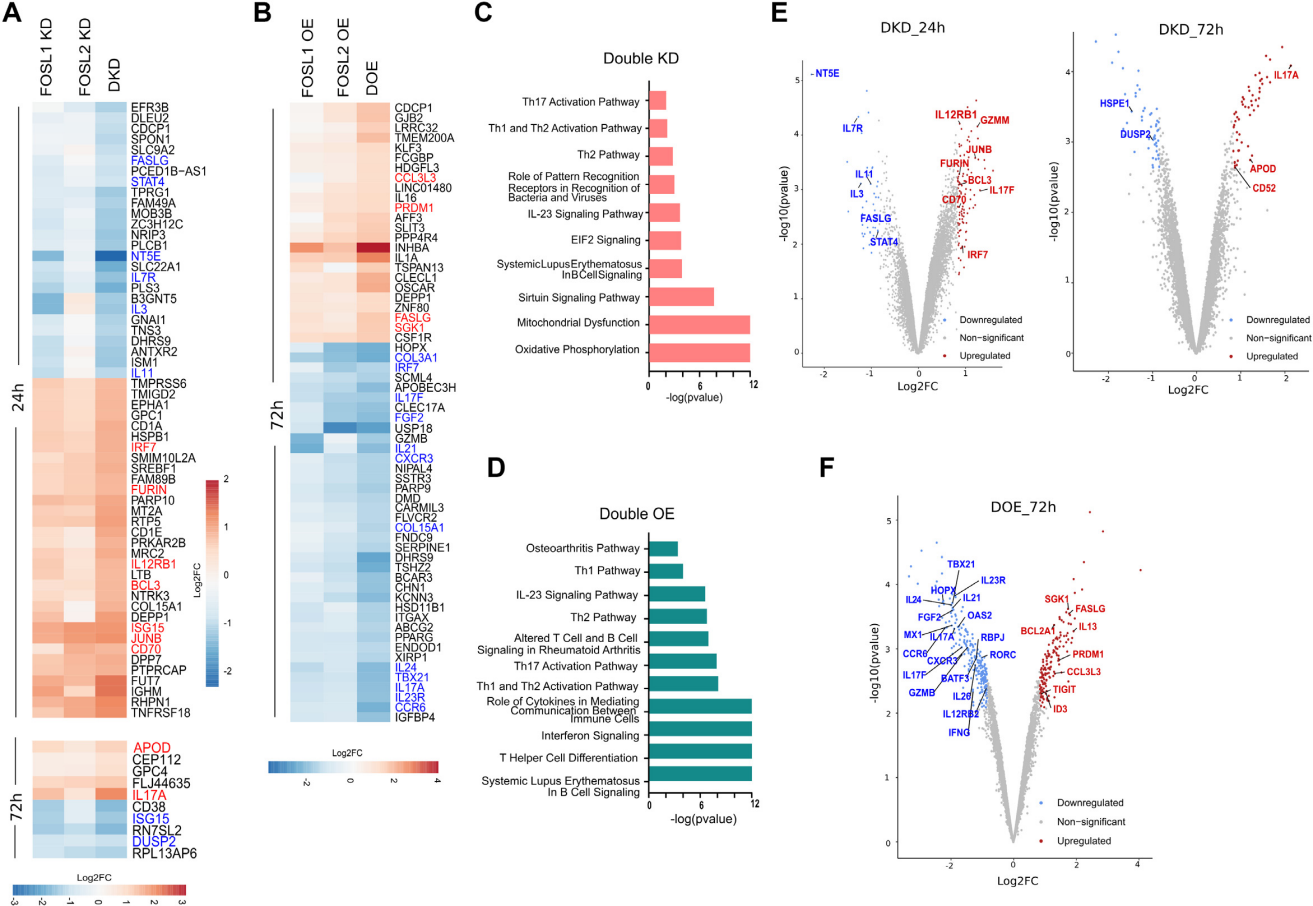
Transcriptional targets co-regulated by FOSL1 and FOSL2. (**A, B**) Heatmap in panel A shows the DE genes that are more profoundly altered in FOSL DKD Th17 cells, as compared to the single KD controls, at 24 h (above) and 72 h (below) of polarization. Panel B depicts the DE genes that show enhanced changes in FOSL DOE Th17 cells, as compared to the single OE controls, at 72 h of polarization. Fold-change (FC) was calculated relative to the respective control conditions (i.e. SCR or GFP). The FDR filtered (≤0.1) DE genes with |FC| ≥ 1.8 in DKD, and |FC| ≥ 2 in DOE, are included in the corresponding heatmaps. Th17-relevant, upregulated genes are depicted in red, and the downregulated ones are in blue. (**C, D**) Ingenuity pathway analysis (IPA) was used to identify signaling pathways that are altered upon FOSL DKD (panel C) or DOE (panel D). The top pathways related to T-cells and immune signaling are selectively shown. (**E, F**) Genome-wide expression analysis of FOSL DKD and DOE Th17 cells. Volcano plots in panel E highlight the Th17-associated transcripts that are differentially expressed upon co-depletion of FOSL1 and FOSL2, at 24 h (left) and 72 h (right) of Th17 polarization. Panel F shows the Th17-associated genes that are differentially expressed upon parallel over-expression of FOSL1 and FOSL2, at 72 h of Th17 polarization. Targets with FDR ≤ 0.1 and |FC| ≥ 1.8 have been plotted. Upregulated genes are in red, and the downregulated ones are in blue (for extended list of DKD and DOE targets, refer to Supplementary Figure S3A, B).

To further identify the co-regulated targets, the fold-changes for the affected genes were compared in KD versus DKD and OE versus DOE conditions. At least 50 of the DE genes in KD and OE each, showed more pronounced expression changes when both factors were simultaneously perturbed (Figure 3A, B). These included key Th17-marker genes such as *IL17A, IL17F, IL23R* and *CCR6*, all of which were negatively regulated. Similarly, other factors known to modulate Th17-cell responses (*FASLG* (53,54), *IL7R* (55), *NT5E* (56–59), *STAT4* (60–63), *CD70* (64,65), *PRDM1* (22), *FGF2* (66), *DUSP2* (67), *IL12RB1* (68), *IL11* (69–71), *IL24* (72–74) and *IRF7* (75)*)*, were also found to be co-regulated. Nonetheless, we discovered a few lineage-associated genes (*IL21, USP18, GZMB, IL3*, and others) that were altered by FOSL1 and FOSL2 in a distinctive fashion (Figure 3A, B). This suggests that apart from their coordinated roles, these factors also independently guide Th17-gene networks.

Since co-perturbation affected a wider range of Th cell-related genes, we hereby focused on analyzing the DKD and DOE datasets (extended list of DE genes in Supplementary Figure S3A, B). Ingenuity pathway analysis (IPA) indicated that the genes perturbed by FOSL KD/OE were involved in mitochondrial dysfunction, oxidative phosphorylation, T-helper cell differentiation, IL-23 signaling, Interferon signaling, Th1/Th2/Th17 activation, and autoimmune processes (RA, systemic lupus erythematosus (SLE)) (Figure 3C, D). It is possible that FOSL factors have time-dependent functions since the DKD targets at 24 h and 72 h of polarization show a limited overlap (Supplementary Figure S3C). This could be due to multiple factors, one of them being the distinct expression profiles of human Th17 cells at the two time points (8,9). It could also be a result of changes in KD efficiency with time as the transient KD is at its highest at 24 h, where after it dilutes as the cells proliferate.

The regulatory networks guiding Th17 function are largely unexplored in human, and only a handful of genes have been characterized on this front. In a recent study however, Hu *et al.* used the global murine Th17 transcriptome along with other autoimmune studies to shortlist a ‘HuTh17 codeset’, which encompasses 418 genes associated with human Th cell differentiation and activation (76). We found 53 of these codeset genes, including 25 Th17-related targets (Supplementary Table S10), to be differentially expressed upon FOSL co-perturbation (FDR ≤ 0.1, |FC| ≥ 1.8 in DKD or DOE). We next compared the FOSL gene-targets with STAT3, which is the critical transcription factor priming Th17 development (43,50). Out of the 42 genes known to be strongly regulated by STAT3 in human (43), we found 24 to be significantly affected in our FOSL RNA-seq data. Importantly, 22/24 genes were controlled by FOSL factors in a manner opposite to STAT3 (including *RORA, IL12RB2, GZMB, CCR6, IL24, IL23R, HOPX, PTGER2, NR4A2*) (Supplementary Table S10, based on DOE). FOSL also negatively affected the signature genes of (human) RORγT *(CCR6, IL26, CTSH, PPARG, IL17F, IL17A) (**77**)*, which is a master regulator of Th17-fate. In addition to the above, FOSL proteins potentially repressed genes such as *FGF2* (66), *IL21* (78), *JUNB* (79,80), *CD70* (64,65), *IL12RB1* (68), *CD52* (81,82), *RBPJ* (83), *OAS2, MX1* and *ISG15* (84), which either promote the Th17 lineage or serve as autoimmune markers in human or mouse (Figure 3E, F; Supplementary Figure S3A, B). At the same time, FOSL enhanced the expression of genes that inhibit Th17 function and autoimmune-development (*IL13* (85), *IL7R* (55), *PRDM1* (22), *DUSP2* (67), *BCL2A1* (86), *ID3* (87), *TIGIT* (88) and *NT5E* (56–59)*)* (Figure 3E, F; Supplementary Figure S3A, B). These findings thus imply that FOSL1 and FOSL2 negatively influence early stages of Th17 signaling in human.

Next, we used immunoblotting and flow cytometry analysis to validate the expression changes of some of the FOSL RNA-seq targets that had relevance to Th17-function. CCR6 is a chemokine receptor that is preferentially induced upon human Th17 differentiation (89). Flow cytometry analysis found the surface expression of CCR6 to be downregulated upon FOSL DOE and upregulated upon DKD (Supplementary Figure S3D). NT5E or CD73 is a 5′-ectonucleotidase known to resolve uncontrolled inflammation (57,90). A positive correlation was reproducibly detected between FOSL and NT5E expression, which hints at their interlinked participation in keeping inflammatory responses in check (Supplementary Figure S3E and Supplementary Figure S4A). Further, co-depletion of FOSL1 and FOSL2 altered protein-level expression of CD70, STAT4, APOD and JUNB, all of which have reported links to Th17 differentiation (9,60,63–65,79,80,91) (Supplementary Figure S3E and Supplementary Figure S4B-D). We additionally performed qRT-PCR analysis (Supplementary Figure S5A, B) to validate more of the DKD and DOE targets that were associated with Th cell lineage specification (*GATA3, MIAT, IFNG, IL23R, RORC, TBET, IL3* and *STAT4*). Collectively, the above results strengthen the role of FOSL proteins in human Th17 regulation.

### FOSL1 and FOSL2 share occupancy over their co-regulated gene-targets

**Figure 4.**
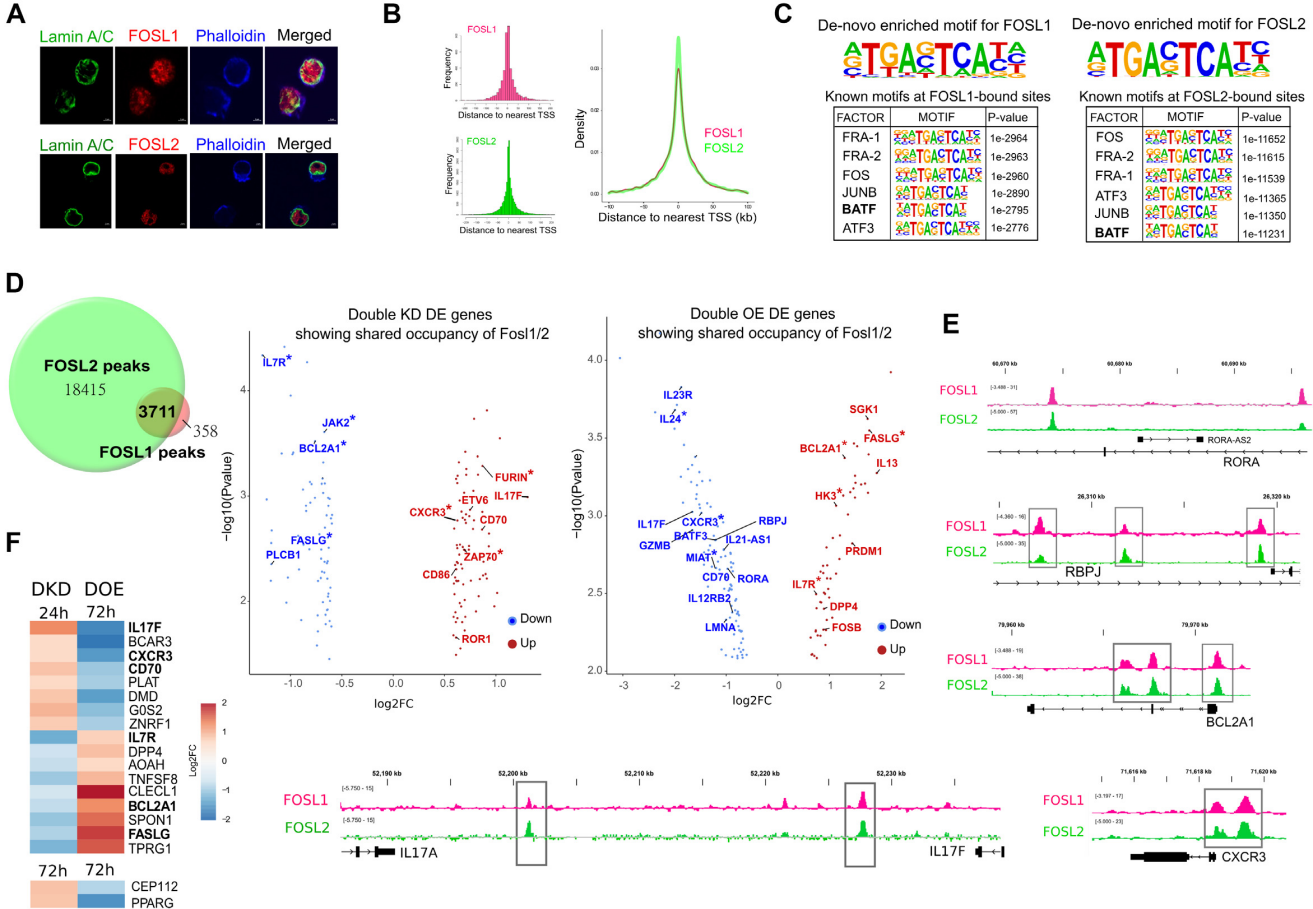
Genome-wide occupancy profile of FOSL proteins in human Th17 cells. (**A**) Immunofluorescence images showing nuclear localization of FOSL1 (red, above panel) and FOSL2 (red, below panel) in 72 h-polarized Th17 cells. Lamin A/C (in green) marks the nuclear periphery, whereas phalloidin (in blue) stains the cytoplasmic actin. (**B**) ChIP-seq analysis was performed for FOSL1 and FOSL2 using 72 h-cultured Th17 cells. Figures on the left show distribution of FOSL1 and FOSL2 binding sites relative to the position of the closest transcription start site (TSS). TSS is defined to be at position zero. Figure on the right shows an overlay of the peak distribution profiles of the two factors. (**C**) The topmost consensus sequences for FOSL1 and FOSL2 genomic binding were identified using *de-novo* motif enrichment analysis by Homer. FOSL1 (left) and FOSL2 (right) peaks were further enriched for known TF motifs, and the top motifs identified by Homer are shown. Peaks with IDR *p* < 0.01 were used for motif discovery. (**D**) ChIPpeakAnno was used to determine the overlap in the genomic binding sites of FOSL1 and FOSL2 (overlap represents peaks sharing 200 bp or more). Genes neighbouring to these overlying sites and differentially expressed under DKD or DOE conditions (FDR ≤ 0.1, |FC| ≥ 1.5) were assigned as the shared-direct targets of FOSL1 and FOSL2. Adjoining volcano plots show the logarithmic fold changes for selected shared targets (DKD (left); DOE (right)). Downregulated genes are in blue, and upregulated ones are in red. Targets with FOSL occupancy over promoter regions (5-kb window around TSS) are marked with asterisk. (**E**) Integrative Genomics Viewer (IGV) track snapshots show the binding overlap of FOSL1 and FOSL2 over selected Th17-associated genes. (**F**) Heatmap depicts the shared direct targets that show opposite expression changes in FOSL DKD and DOE conditions, at the indicated time points of Th17 differentiation. Th17-relevant genes have been highlighted.

AP-1 proteins function as transcriptional regulators by directly binding to the target gene loci. To elucidate the global occupancy profiles of FOSL1 and FOSL2 in human Th17 cells, we performed chromatin immunoprecipitation, followed by sequencing (ChIP-seq) analysis. Since these factors portray cell type-specific cellular localization (92,93), immunofluorescence analysis was first used to confirm their predominant nuclear profile in Th17 cells (Figure 4A, Supplementary Figure S6A). Results from our recent study validate this trend (94), wherein immunoblotting was used to analyse FOSL expression in subcellular fractionated Th17-cell lysates (nuclear and cytoplasmic).

Our ChIP-seq analysis identified 22,127 peaks for FOSL2 and 4,088 peaks for FOSL1 (with irreproducible discovery rate (IDR) significance of < 0.01) (Supplementary Table S4). In agreement with previous findings (95–97), a large fraction of these peaks covered intergenic/intronic regions, therefore suggesting that these factors control gene-expression through distal regulatory elements (Supplementary Figure S6B). The peak distribution profiles of the two proteins are shown in Figure 4B. Interestingly, known FOSL2-binding motifs were detected within FOSL1 peaks and vice-versa, which underscores their propensity to bind to overlapping regions (Figure 4C). De-novo motif enrichment analysis further identified the consensus DNA-binding sequences of these TFs (Figure 4C).

FOSL1 and FOSL2 are reported to co-occupy selective gene targets in breast cancer cells (51,52). We examined if a similar paradigm exists in human Th17 cells, which potentially mediates the coordinated roles of these factors. Our genome-wide occupancy analysis revealed 3,711 binding sites to be common between FOSL1 and FOSL2 (Figure 4D, Supplementary Table S4). Interestingly, more than 150 genes in nearest vicinity of these shared sites were co-perturbed in our transcriptome analysis (DKD and DOE each) (FDR ≤ 0.1 with |FC| ≥ 1.5) (Figure 4D). These were assigned as the shared-direct targets (Supplementary Table S4) of FOSL1 and FOSL2, and included multiple Th17-relevant genes that were either activated (*IL13* (85), *IL7R* (55), *JAK2* (98), *BCL2A1*(86), *FASLG* (53,54,99), *PRDM1* (22)*)* or repressed (*IL17F, IL23R, FURIN* (100), *RBPJ* (83), *CXCR3* (101–103), *MIAT* (104), *IL24* (72–74), *ETV6* (2), *ZAP70* (105), *RORA)*. Supplementary Figure S6C shows the top (selected) immune signaling pathways enriched for the shared targets. Integrative genomics viewer (IGV) tracks in Figure 4E illustrate the binding overlap of FOSL factors over specific Th17 gene-targets. To further identify the genes that are strongly co-regulated, we focused on the subset of FOSL direct targets that showed contrasting expression changes in DKD and DOE (Figure 4F). We found 19 such genes, including Th17-specific factors such as *IL17F, CXCR3* (101–103), *FASLG* (53,54), *IL7R* (55), *BCL2A1* (86) and *CD70* (64,65). In addition, two of the direct targets (GPR87 and S10082) showed similar expression changes in DKD and DOE.

An interesting candidate among the shared targets was *PRDM1 (*or *BLIMP1)*, an inhibitor of Th17-differentiation (22), that was directly-bound and positively regulated by FOSL1 and FOSL2. We found this to corroborate with previous findings in the field (106). We additionally observed that only one-third of the shared targets showed FOSL occupancy over putative promoter regions (5-kb window around the TSS) (Supplementary Figure S6D, asterisked in Figure 4D). The remaining majority were bound over intronic or intergenic sites. This highlights the fact that FOSL1 and FOSL2 co-regulate Th17-specific genes presumably by occupying enhancer or silencer elements in the genome.

### FOSL proteins and BATF co-localize over key Th17 genes and regulate their expression in an opposite fashion

Genomic co-occupancy is a distinguished feature of FOS, JUN and ATF family members (2,79,107). Considering this, the FOSL1 and FOSL2 ChIP peaks were screened for the presence of other (known) TF-motifs (Supplementary Table S4). Our analysis revealed binding motifs for BATF, JUNB, FOS and ATF3, among the top identifications (Figure 4C). A former study suggested an antagonistic relationship between BATF and FOSL2, during murine Th17-differentiation (2). We aimed at verifying whether BATF similarly interplays with FOSL1 and FOSL2, while regulating human Th17 responses.

**Figure 5.**
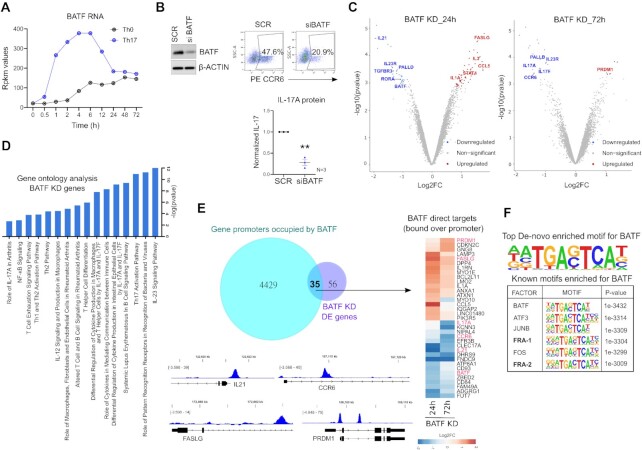
Loss of BATF impairs Th17 differentiation. (**A**) Rpkm values are plotted for BATF RNA at different time points of activation (Th0) or Th17-differentiation, using our published RNA-seq data (8). (**B**) Immunoblot (left) shows BATF protein levels in SCR versus BATF KD cells, at 24 h of Th17 polarization. Actin serves as loading control. Adjoining flow cytometry plots show percentage of CCR6 positive cells and the graph below shows ELISA analysis for IL-17 secretion in SCR versus BATF KD cells, at 72 h of Th17 polarization. ELISA values were first normalized for cell count (live), and then normalized to SCR control. Graph shows mean ± SEM for three biological replicates. Statistical significance was calculated using two-tailed Student's *t* test (**p < 0.01). (**C**) Volcano plots highlight the significantly upregulated (in red) and downregulated (in blue) genes in BATF-silenced Th17 cells at 24 h (left) and 72 h (right) of polarization (FDR ≤ 0.1, |FC| ≥ 1.8). DE genes with relevance to Th17 function are shown (extended DE gene list shown in Supplementary Figure S7D). (**D**) IPA was used to identify pathways altered upon silencing of BATF in Th17-polarized cells (24 h and 72 h). The top pathways related to T-cells and immune signaling are selectively shown. (**E**) Venn diagram shows the overlap between the genes that are altered upon BATF KD and the genes whose putative promoter regions (5-kb window around the TSS) are bound by BATF. The overlapping area represents the promoter-bound regulatory targets of BATF and the adjoining heatmap shows their corresponding expression changes in BATF KD Th17 cells. IGV images illustrate the occupancy of BATF over some of its Th17-associated targets. (**F**) Figure shows the topmost consensus sequence for genomic-binding of BATF, and the top six TF motifs enriched within BATF-bound sites, which were obtained using de-novo motif enrichment analysis by Homer. Peaks with IDR *p* value <0.01 were used for motif discovery.

BATF is a key-modulator of murine Th17 fate (25,108), however, its role in the human counterpart remains unknown. When compared to activation conditions, we found BATF to be consistently upregulated during the course of Th17 differentiation, at both RNA and protein level (Figure 5A, Supplementary Figure S7A). To address its role in human, we used RNAi, where naive CD4^+^ T-cells were nucleofected with BATF-targeting siRNA and further polarized to Th17 phenotype. Loss of BATF significantly reduced both CCR6 and IL-17 expression, at 72 h of polarization (Figure 5B; Supplementary Figure S7B, C). Further bolstering these results, transcriptome analysis of BATF KD cells confirmed the downregulated expression of multiple Th17-marker genes including *IL17A, IL17F, IL23R, CCR6* and *IL21* (Figure 5C; extended list of DE genes in Supplementary Figure S7D, Supplementary Table S5). Additionally, Ingenuity pathway analysis found BATF to alter genes involved in IL-23 signaling, T-helper cell differentiation, Th17 activation, and autoimmune processes (SLE, RA) (Figure 5D).

Next, we examined the global occupancy profile of BATF in Th17 cells by ChIP-seq analysis, which identified a total of 16,479 binding sites (IDR significance < 0.01) (Supplementary Table S5). At least 64 genes in nearest vicinity of these sites were perturbed by BATF in our transcriptome analysis (FDR ≤ 0.1, |FC| ≥ 1.8). These were regarded as the BATF direct targets (Supplementary Table S5). BATF also occupied the promoter regions of more than 4000 genes, 35 of which were regulated by the factor (Figure 5E). Adjoining IGV images in Figure 5E illustrate the occupancy of BATF over promoters of key Th17 genes (*IL21, CCR6, PRDM1 and FASLG*). Further, motif analysis of the ChIP-seq peaks revealed BATF as the topmost known-motif and identified the consensus sequence for its genomic-binding (Figure 5F, Supplementary Table S5).

**Figure 6.**
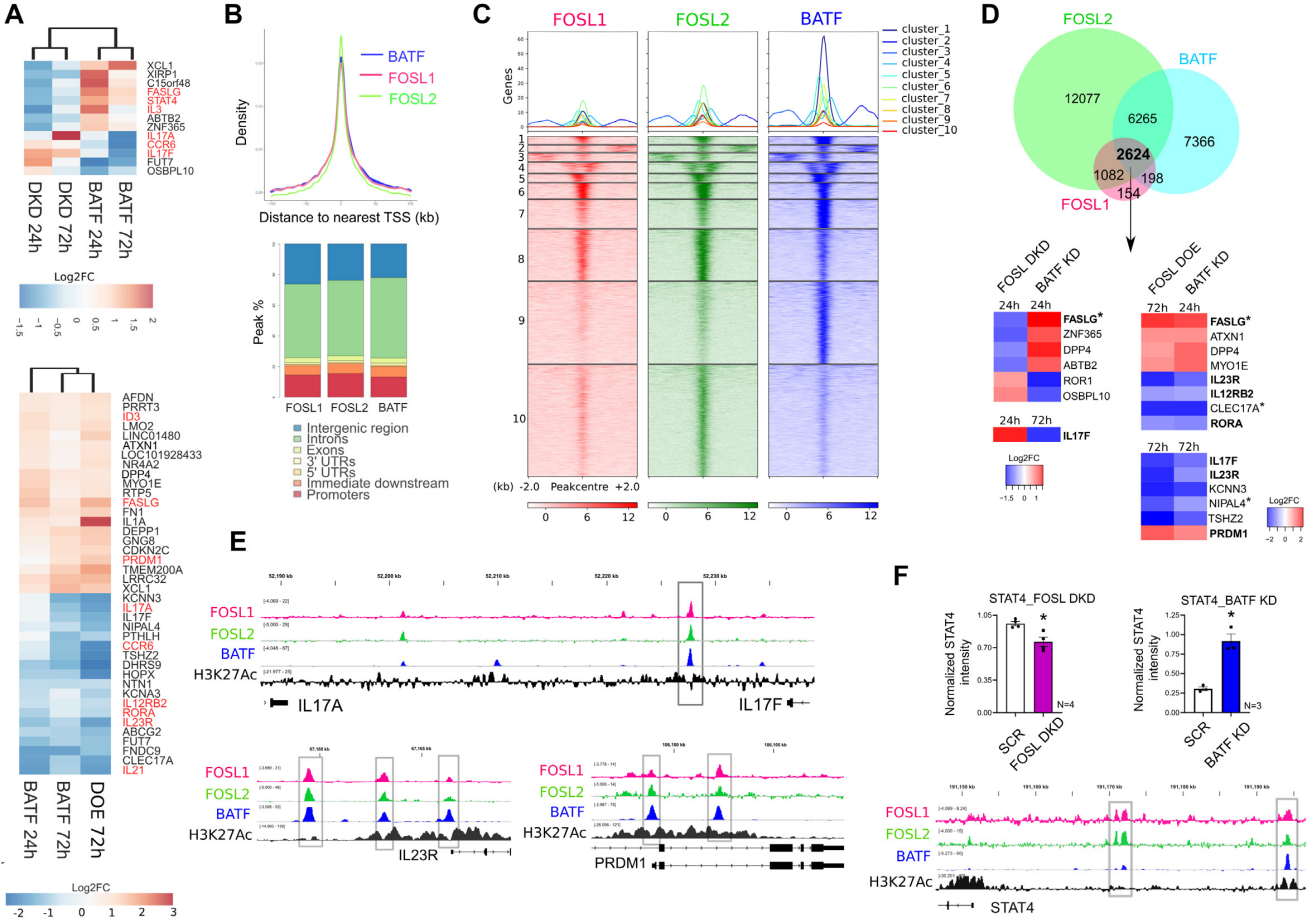
Comparing transcriptional targets and genomic binding sites of FOSL proteins with BATF. (**A**) Heatmap on the top shows logarithmic FC values for the DE genes that show opposite expression changes in FOSL DKD and BATF KD Th17 cells, at the indicated time points of differentiation. Heatmap in the bottom panel depicts the DE genes that show similar expression changes in FOSL DOE and BATF KD Th17 cells. Th17-related genes are highlighted in red. (**B**) ChIP-seq profiles of FOSL1, FOSL2 and BATF in Th17 cells. Graph (above) shows the overlay between the peak distribution profiles of the three TFs. Bar plot (below) depicts peak-annotation results for their identified binding sites. (**C**) Heatmap with k-means clustering shows the ChIP-seq signal intensities ± 2-kb around the centers of the genomic-binding regions of FOSL1, FOSL2 and BATF. (**D**) Venn diagram shows an overlap between the genomic binding sites of FOSL1, FOSL2 and BATF (overlap represents peaks sharing 200 bp or more). Adjoining heatmap depicts Log2FC values for the gene targets that are co-bound and oppositely regulated by FOSL proteins and BATF, at the given time points of Th17 differentiation. Genes showing shared occupancy of the three factors over promoter regions have been marked (*asterisk). Th17-related targets are highlighted. (**E**) IGV track snapshots illustrate the co-localization of FOSL1, FOSL2 and BATF over selected Th17-linked genes. The profile of H3K27ac histone mark around the shared binding sites of the three factors is shown. (**F**) Bar plot depicts immunoblot-based expression analysis of STAT4 in FOSL DKD (left) and BATF KD (right) Th17 cells, cultured for 72 h. Data shows mean ± SEM for three or four biological replicates, as indicated. Statistical significance is calculated using two-tailed Student's *t* test (*p < 0.05). Adjoining IGV track shows the binding overlap of FOSL1, FOSL2 and BATF, flanked by H3K27ac marks near the STAT4 locus.

The above findings collectively establish that BATF positively regulates early Th17 differentiation in human, and thus exhibits functions antagonistic to FOSL proteins. To dissect this antagonism at the level of gene targets, we compared the DE genes for BATF KD and FOSL DKD, and focused on the candidates that were common but regulated in an opposite fashion (Figure 6A, top panel). Likewise, the genes that showed similar expression changes in BATF KD and FOSL DOE were selected (Figure 6A, bottom panel). Based on our analysis, the Th17-lineage defining genes that were negatively regulated by FOSL proteins (*IL17A, IL17F, IL21, RORA, IL23R* and *CCR6)*, were found to be positively regulated by BATF. Alongside, the Th17-repressor genes that were activated by FOSL factors (*PRDM1* (22) and *ID3* (87)), were potentially inhibited by BATF.

To further examine which of the genes regulated in an opposite fashion are also bound by these TFs, we compared the ChIP-seq profiles of the three proteins. An overlay of their peak distribution plots (top) and a comparative peak annotation plot (bottom) is shown in Figure 6B. In addition, mapping of the ChIP-seq signal intensities ± 2-kb around the centers of the genomic-binding regions of FOSL1, FOSL2 and BATF, evidently demonstrated the unanimity in their DNA binding pattern (Figure 6C). Their individual sites were further clustered; where clusters 1, 7 & 9 showed higher enrichment of BATF, whereas cluster 8 depicted greater signal densities for FOSL1 and FOSL2. We then used the R package ChIPpeakAnno (37) to determine the exact overlap in their binding sites, and discovered a total of 2,624 sites to be common (Figure 6D, Supplementary Table S6). A total of 17 genes in the nearest vicinity of these common binding sites were found to be regulated by FOSL and BATF in the opposite direction (with FDR ≤ 0.1, |FC| ≥ 1.5) (Figure 6D heatmap). These included six key genes associated with Th17 cell-function (*RORA, IL17F, IL23R, PRDM1, FASLG, IL12RB2*). We thus propose that BATF and FOSL contextually or competitively bind to a common set of Th17-related genes, and oppositely regulate lineage-specification. Our analysis further indicated that BATF, FOSL1 and FOSL2 primarily occupy regulatory DNA elements distal to the promoter (Figure 6B, bottom) for altering the expression of their target genes (Figure 6D, non-asterisked genes).

Many AP-1 TFs show co-localized binding on genomic regions that have enhancer marks (H3K4me1/H3K27ac). Such regulatory modules are known to drive cellular differentiation and disease-associated functions (96,109). To scrutinize our study on this front, we examined the ChIP peaks of FOSL1, FOSL2 and BATF for H3K27Ac marks (a transcriptionally permissive histone modification found on active enhancers and promoters) by using a published human Th17 dataset (38) (GSE101389). IGV tracks in Figure 6E illustrate how H3K27ac flanks the shared binding sites of these AP-1 factors, in the vicinity of their direct targets *(IL17A/F, IL23R* and *PRDM1*). An identical pattern was observed upstream of the human *STAT4* locus (*IGV image*, Figure 6F). Despite its well-established role in Th1/Th17 differentiation and disease (61–63,110,111), STAT4 has not been studied in the context of non-pathogenic human Th17 responses. We thereby checked the effect of FOSL and BATF on STAT4 expression, by immunoblot analysis. Loss of BATF upregulated STAT4 levels, whereas co-depletion of FOSL1 and FOSL2 reduced it (Figure 6F; Supplementary Figure S7E). This suggests a potential link between the AP-1 proteins and STAT4 during early Th17-regulation.

### FOSL1, FOSL2 and BATF exhibit common interacting partners in Th17 cells

FOS, JUN and ATF TFs are known to share some of their binding partners. This possibly creates molecular competition, which is reported to mediate functional antagonism between specific members of the AP-1 family (24,112–114). BATF for instance, competes with FOS proteins for partnering with JUNB, which allows it to negatively influence FOS activity (113). To address if a similar mechanism facilitates the BATF-FOSL antagonism in our study, we primarily checked if these factors have common interactors.

**Figure 7.**
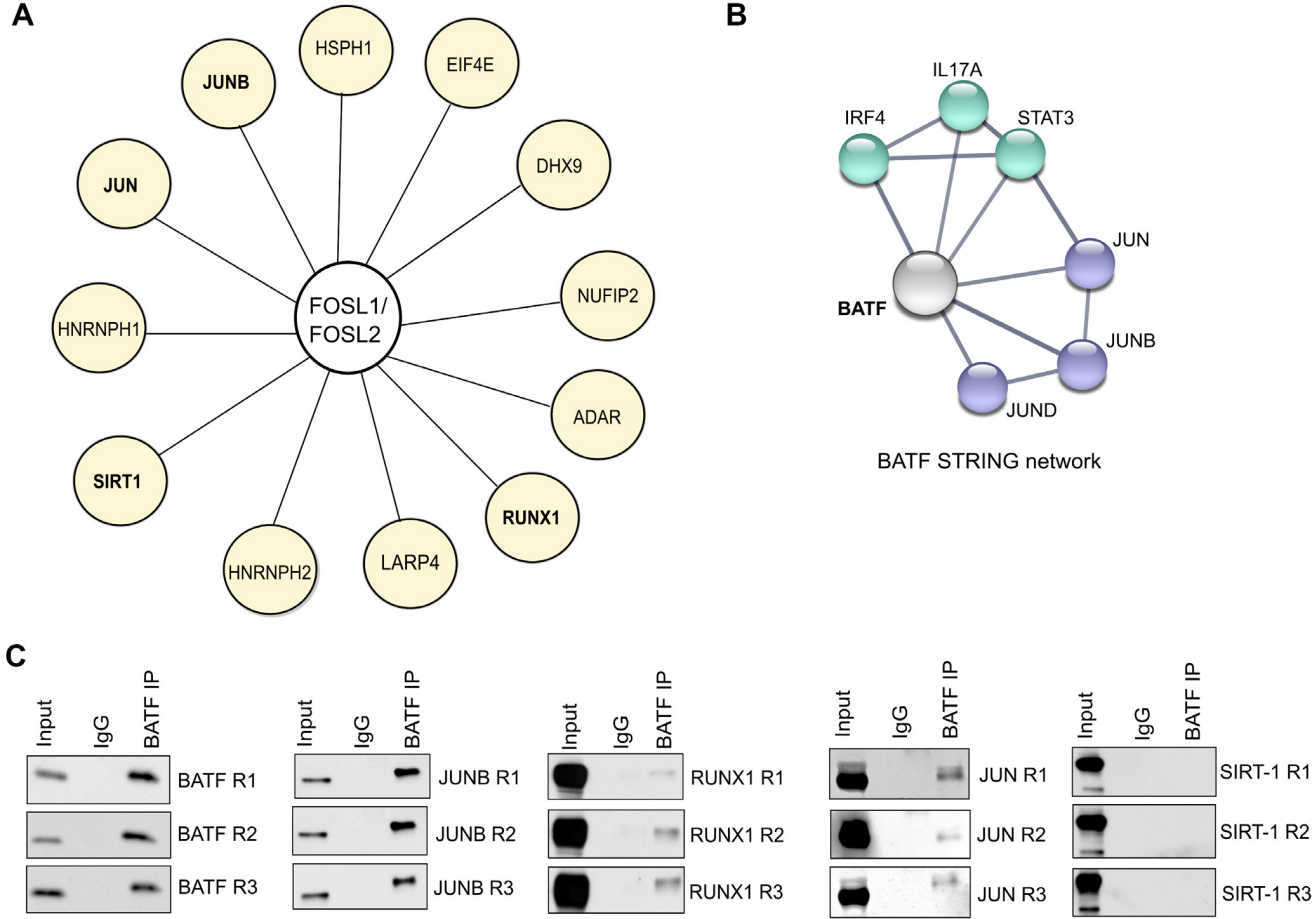
BATF and FOSL proteins show common interacting partners in Th17 cells. (**A**) Figure illustrates the common binding partners of FOSL1 and FOSL2 in Th17 cells (72 h), based on data acquired from our recent study (94). Interactors having reported roles in T-cell function are shown. (**B**) STRING network analysis of human BATF. Width of lines between the nodes indicate confidence values for each protein-protein association. Interactions with a minimum score of 0.7 are shown (high confidence). (**C**) BATF was immunoprecipitated using 72 h-polarized Th17 cultures. Immunoblotting was then used to analyse its interaction with selected (shared) binding partners of FOSL1 and FOSL2 (JUNB, SIRT-1, JUN and RUNX1). Data is shown for three biological replicates. Immunoblot for BATF confirms immunoprecipitation of the factor.

The interactomes of FOSL proteins in human Th17 cells were recently uncovered in our study where we used a global proteomics approach (94). Here, FOSL1 or FOSL2 was immunoprecipitated and the co-precipitated putative interactors were identified by liquid chromatography-tandem MS (LC-MS/MS). Interestingly, the two factors were found to share a total of 29 binding partners, many of which regulate T-cell signaling processes (RUNX1, SIRT1, EIF4E, JUN, JUNB, ADAR, NUFIP2, HSPH1, IFI16, HNRNPH1/2, LARP4 and DHX9) (shown in Figure 7A). Of these, JUN TFs are already reported to interact with BATF in other studies (BATF STRING network, Figure 7B).

To verify these interactions in human Th17 cells, we immunoprecipitated BATF from 72 h-polarized Th17 cultures. Immunoblot analysis was then performed to check its interaction with JUN (JUN, JUNB) as well as other FOSL partners with Th17-relevance (RUNX1 and SIRT-1) (16,79,80,115–117). We found BATF to reliably associate with JUN, JUNB and RUNX1 (Figure 7C). Intriguingly, previous studies have found these proteins to perform context-specific roles based on their choice of binding partner (24,116). This implies that BATF and FOSL potentially compete for common interacting partners, and differentially orchestrate human Th17 responses. Notably, STAT3 and IRF4, which form pioneering complexes with BATF in mouse Th17 cells (2,118), showed no interaction with it in our study (Supplementary Figure S7F).

### Multiple disease-linked SNPs enriched within the genomic binding sites of FOSL1, FOSL2 and BATF affect the ability of these factors to bind DNA

Functional analysis of data from genome-wide association studies (GWAS) has revealed that SNPs linked to disease phenotypes can alter binding sites of key TFs (42). The presence of a SNP can abrogate or enhance TF occupancy, which might subsequently influence gene-expression profiles (43). Interestingly, 90% of the disease-linked SNPs are reported to occur within non-coding genomic regions (119), which also appear to accommodate a major fraction of the TF ChIP peaks in our study. With this in view, we sought to determine if the genomic-binding sites of FOSL1, FOSL2 and BATF harbour any autoimmune-associated SNPs that could disrupt the occupancy of the respective factors.

**Figure 8.**
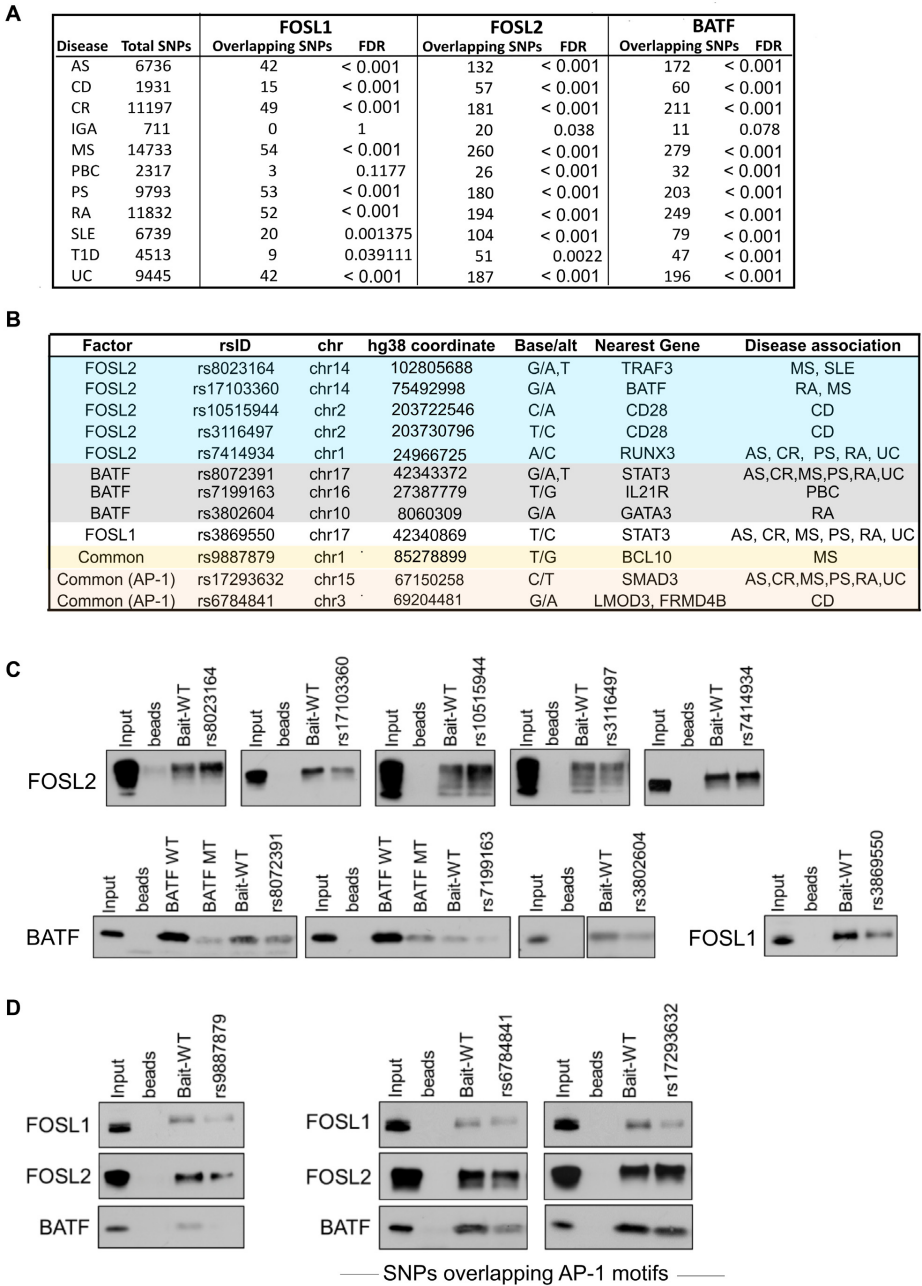
SNPs associated with autoimmune diseases localize within the genomic binding sites of FOSL1, FOSL2 and BATF. (**A**) Enrichment of disease-associated SNPs (or their proxies in Caucasian populations) within FOSL1, FOSL2 and BATF genomic-binding sites, relative to random sets of background SNPs. (**B**) SNPs relevant to the study were shortlisted (Supplementary Table S8). Of these, the SNPs that were functionally validated in DNA-affinity precipitation assays are shown. (**C, D**) DAPA followed by immunoblot analysis shows the SNPs that alter the binding of FOSL1, FOSL2 or BATF to their genomic sites (identified by ChIP-seq analysis). Wildtype (WT) oligonucleotides containing the binding motifs of these TFs (at different genomic loci), and mutant oligonucleotides harbouring a SNP within the binding motif, were used as baits for pull-down of the corresponding AP-1 factor from 72 h Th17-polarized cell lysates. For experimental controls, an oligonucleotide with a conserved binding sequence for BATF (BATF WT), and the corresponding mutated sequence which is known to disrupt BATF occupancy (BATF MUT) were used. Panel C includes SNPs affecting the binding of either FOSL1, FOSL2 or BATF. Those SNPs at the common binding sites of the three factors which also alter the binding affinities for all of them are shown in panel D. The common SNPs harboured within consensus AP-1 motifs are labelled. Data is representative of three biological replicates.

We used the NHGRI-EBI GWAS catalogue from Caucasian populations to primarily query SNPs with reported links to 11 different autoimmune phenotypes (ankylosing spondylitis (AS), celiac disease, Crohn's disease (CD), IgA immunodeficiency, MS, primary biliary cholangitis, psoriasis (PS), RA, SLE, type I diabetes and ulcerative colitis (UC) (Figure 8A). Upon intersecting these with the TF peaks identified in our study, we detected 114, 571 and 573 disease-linked SNPs (and their proxies) within FOSL1, FOSL2 and BATF binding sites, respectively (Supplementary Table S7). Importantly, the genomic binding regions shared between the three factors harboured as many as 64 disease-associated SNPs (Supplementary Table S7).

We further shortlisted the SNPs relevant to our study by screening for the ones that overlap with the TF binding sites in the vicinity of Th17-related genes (Supplementary Table S8). Additionally, the SNPs that were common across the three factors and harboured within canonical AP-1 motifs were listed (Supplementary Figure S8). DNA affinity precipitation assay (also known as DAPA) was then performed to determine if any of these SNPs affect the ability of FOSL1, FOSL2 or BATF to bind DNA. In this assay, we designed wildtype (WT) oligonucleotide probes containing binding motifs of FOSL1, FOSL2 or BATF (at different genomic loci), and mutant (MUT) oligonucleotides with a SNP in the same binding motif. Using these as baits, the corresponding AP-1 factor was precipitated from Th17 cell lysates (72 h) and the pull-down protein was analysed by immunoblotting. We then evaluated if a given AP-1 factor showed differential binding to the WT and the MUT probe.

DAPA analysis of selected SNPs (Figure 8B) revealed important changes in the binding propensities of the three TFs. For instance, we detected altered binding of FOSL2 to mutant oligonucleotides harbouring the following five SNPs: rs8023164 (MS and SLE), rs17103360 (MS and RA), rs10515944 (CD), rs3116497 (CD) and rs7414934 (AS, CR, PS, RA, and UC) (Figure 8C; Supplementary Figure S9A, S10A). These SNPs appeared to occur in the regulatory regions that are neighbouring to *TRAF3, BATF, CD28* and *RUNX3 genes*, which could be potential targets of FOSL2. Interestingly, TRAF3 is reported to enhance T-cell activation (120), restrain IL-2 dependent generation of thymic Tregs (121) and impair IL-17R proximal signaling (122). BATF is a well-known regulator of Th17 responses (25), whereas low CD28 co-stimulation has been found to promote Th17-development (123). Furthermore, while RUNX transcription factors are recognized modulators of Th17-fate (124), RUNX3 in particular, is found to be elevated in CD4^+^ T-cells of PS patients. Notably, loss of RUNX3 impairs Th17 and Th22 differentiation, both of which are required for the pathogenesis of psoriasis (125). Our findings thus suggest that specific SNP mutations alter the ability of FOSL2 to bind to target regulatory DNA elements near important Th17-signaling genes.

We similarly identified three SNPs for BATF (near *IL21R, GATA3* and *STAT3*) and one for FOSL1 (near *STAT3*), which when introduced within the corresponding TF motif, significantly disrupted occupancy of these factors (Figure 8C, Supplementary Figure S9A, S10A). IL-21 and STAT3 positively regulate Th17 cell programs (43,68,78,126,127), whereas GATA-3 is a master regulator of Th2-fate, which also constrains Th17-mediated pathology (128,129). Interestingly, a BCL10-proximal SNP rs9887879, which overlaps the shared binding sites of FOSL1, FOSL2 and BATF, reduced the DNA-binding affinities for all of them (Figure 8D; Supplementary Figure S9B, S10B). BCL10 suggestively regulates Th17 function as a part of a signaling complex (130). It is a key component of the Carma1-Bcl10-Malt1 complex that is essential for pathogenic Th17 responses (131). We additionally validated the functional effects of two other SNPs—rs17293632 near *SMAD3* (linked to AS, CR, MS, PS, RA and UC), and rs6784841 near *LMOD3/FRMD4B* genes (linked to CD) (Figure 8D; Supplementary Figure S9B, S10B). These occurred within consensus AP-1 motifs at the shared genomic-binding regions of the three factors, and altered the binding propensities for all of them. The ability of the above-mentioned SNPs to perturb genomic occupancy of these TFs could trigger changes in their Th17-regulatory roles, thereby facilitating the development of multiple autoimmune phenotypes.

## DISCUSSION

FOS and ATF proteins are established regulators of proliferation, differentiation and apoptosis in many cancers. Their involvement in specification of Th cell lineages, however, has been investigated only recently. Th17-specific AP-1 networks have been mostly studied using mouse models. Taking into account the recently studied heterogeneity between human and mouse Th17 cells (9), we used human T-cells to verify the roles of FOSL1, FOSL2 and BATF during early Th17-regulation.

AP-1 factors cooperate with each other to drive gene-expression programs (24). FOS proteins, in particular, exhibit functional redundancy that allows them to compensate for the loss of each other (24,132,133). The present study reveals that the individual perturbation of FOSL1 or FOSL2, only modestly alters Th17 cell-identity. Disrupting them in parallel, however, causes additive changes in gene-expression. Intriguingly, co-depletion or dual over-expression of the two factors coordinately affects several Th17-related genes (*IL17A, IL17F, NT5E, CCR6, IL7R, IRF7, BCL2A1, DUSP2, PRDM1, IL21, JUNB, IL23R, CXCR3, IL12RB1, CD52, TIGIT, ID3*). Our findings thus confirm that these paralogs jointly instruct the initial stages of human Th17 cell-differentiation.

Previous studies in mouse indicate that FOSL2 suppresses Th17-responses, yet promotes the expression of genes involved in sustenance of the lineage (2). Our results, however, portray a different scenario. Selected genes associated with Th17 maintenance/survival (*Il23r, Il12rb1* and *Il21*) that were activated by FOSL2 in mouse (2), were in fact inhibited by it in the human counterpart. This implies that although FOSL2 similarly represses Th17 cell-effector genes in the two species, its involvement in parallel signaling networks may differ in human and mouse. FOSL proteins were additionally found to co-influence multiple genes involved in the development of other T-helper cell fates, including *TBX21, GATA3, IFNG, FURIN, BATF3, BCL3, IL12RB2, HOPX* and *IL13*. For instance, FOSL1 and FOSL2 negatively-regulated Th1-lineage genes (*TBX21* (134,135), *IFNG* (136), *BCL3* (137), *IL12RB2* (138), *HOPX* (139)), while promoting the expression of Th2-specific factors (*GATA3* (128), *IL13* (140)*)*. It however remains to be determined whether FOSL proteins truly restrain Th17 responses by modulating Th-cell lineage diversification.

Though murine studies have examined the molecular networks that drive the transition from homeostatic- to pathogenic-Th17 fate, this switch is not well characterized in human. Our transcriptome analysis revealed FOSL1 and FOSL2 to suppress multiple genes that positively correlate with Th17-pathogenicity, including *GZMB* (141), *IL23R* (142,143), *RBPJ* (83), *IFN-γ* (144,145) and *TBX21* (124,141,146). In addition, FOSL factors downregulated *IL-26* expression, a cytokine that marks inflammatory Th17-populations in patients suffering from Crohn's disease (147). They also inhibited the expression of *FGF2*, which coordinates with IL-17A to drive autoimmune arthritis (66). These findings suggest that FOSL proteins could help in retaining the protective nature of Th17 cells, under conditions of adversity. Furthermore, they affected the expression of several receptors/ligands including *CCL3L3* (148), *CCL4* (149), *CXCL8* (150), *CXCR3* (101–103) and *CCR6* (89), that govern the migration of inflammatory T-cells in autoimmune phenotypes. Follow-up investigation on how FOSL1 and FOSL2 modulate pathogenic Th17-signaling could help define their potential in the treatment of relevant diseases.

STAT3 acts as a master-regulator of Th17-differentiation in both human and mouse (43,63,151–154). We found FOSL proteins to inhibit multiple genes that are known to be activated by STAT3 (such as *RORA, GZMB, IL12RB2, CCR6, IL24, IL23R, HOPX, GBP4, FNDC9*) (43). Despite their opposite roles in controlling Th17-fate, STAT3 positively regulated FOSL expression in human Th17 cells. The existence of a STAT3-based mechanism to induce these Th17-repressors could be explained through a recent study, where STAT3 was found to alternatively drive a negative-feedback loop that limits Th17-mediated tissue damage in human (155).

The functional antagonism between FOSL2 and BATF is well-reported in mouse (2). Our study is the first one to investigate the relationship between these factors in human Th17 cells. Our findings further reveal how BATF function differs from FOSL1 at the level of transcriptional regulation, which has not been addressed before. BATF and FOSL factors were found to directly bind and oppositely-regulate key Th17 marker genes (*IL17A, IL17F, IL23R, CCR6, IL21*), along with other candidates that are associated with the lineage (*IL3, STAT4, FASLG, PRDM1, IL12RB2* and *RORA*). A cardinal target among these was *FASLG*, which is a crucial regulator of apoptosis (53,54,99). We found its expression to be driven by FOSL proteins and inhibited by BATF. Responsiveness to FAS-signaling contextually varies for pro-inflammatory and anti-inflammatory cells, and is reported to subsequently decide whether autoimmunity develops (53,156,157). Insights on AP-1-governed FAS networks could thus hold significance in disease-biology.

Our analysis found BATF to regulate many Th17-lineage genes by occupying their promoter-regions. However, the BATF-bound sites that co-localized with FOSL near their oppositely regulated targets, mostly occurred within intergenic or intronic elements. In mouse Th17 cells, several AP-1 factors are known to co-bind their consensus motifs within the intergenic regions of *Il17a/f* loci (79). This paradigm appears to be conserved, since we discovered FOSL1, FOSL2 and BATF to similarly intersect over the corresponding gene loci in human Th17 cells. Many studies including ours, indicate that such binding convergence occurs over enhancer landscapes (96,107,158), which potentially govern lineage-identity and plasticity of T-helper cell fates (159,160). Nonetheless, it remains to be understood whether the differentiation-induced epigenomic changes are guided by these AP-1 factors in human T-cells.

The shared genomic occupancy of FOSL and BATF warrants further investigation. Although the overlap in their ChIP peaks suggests co-occupancy or competitive binding, additional experiments are required to ascertain the precise mode of their action. For instance, competitive-binding of these TFs could be confirmed through gene-perturbation approaches, provided that either of these factors show an enhanced occupancy in absence of the other. Such findings have previously been reported for JUNB and JUND, which also exhibit functional antagonism in Th17 cells (79). Although our results highlight the BATF-FOSL interplay in regulation of Th17-effector functions, follow-up studies are required to address their crosstalk in the very early signaling events. BATF acts as a pioneer factor that mediates nucleosomal clearance at lineage-associated loci, during the induction of T-cell differentiation (161). FOSL factors, however, have been poorly studied on this font. Studying the temporal dynamics of their functions is a pre-requisite to understanding the interlinked roles of these AP-1 factors.

We identified hundreds of autoimmune disease-associated SNPs within the genomic-binding sites of FOSL1, FOSL2 and BATF. An ongoing study from our lab further revealed that a large fraction (60–80%) of these binding sites overlap with Th17-specific enhancers. Remarkably, the enhancer-specific binding regions of FOSL1, FOSL2 and BATF harboured as many as 100, 470 and 478 disease-linked SNPs, respectively (unpublished data). Notably, over 50 of these SNPs were common across the three factors. These single-base changes could alter AP-1 function at the distal regulatory elements that dictate the Th17 gene expression program. Furthermore, we found some of the disease-SNPs to affect the binding abilities of FOSL1, FOSL2 and BATF in *in vitro* DNA-binding assays. Regardless, these effects need to be confirmed by generating SNP knock-in clones and examining the *in vivo* influence of these SNPs on the genomic occupancy of these factors, when compared to reference clones. This could help in studying the subsequent changes in the Th17-regulatory roles of FOS and ATF proteins, which potentially associates with the development of autoimmunity.

## DATA AVAILABILITY

The RNA-seq and ChIP-seq datasets supporting the conclusions of this article are submitted to GEO with the accession numbers - GSE174809 and GSE174810. All other data is available in the main text or as a part of the Supplementary information.

## Supplementary Material

gkac256_Supplemental_Files
