# Supplementary material for: A systematic comparison of FOSL1, FOSL2 and BATF-mediated transcriptional regulation during early human Th17 differentiation
Source: Nucleic Acids Res. 2022 May 3;50(9):4938–58. doi: 10.1093/nar/gkac256 (PMC9122603; doi:10.1093/nar/gkac256)
Supplement: gkac256_Supplemental_Files [file gkac256_supplemental_files.zip › NAR Supp information_Final.pdf]

SUPPLEMENTAL INFORMATION

A systematic comparison of FOSL1, FOSL2 and BATF-mediated transcriptional regulation during early human Th17 differentiation

Ankitha Shetty[1,2,3,†], Subhash Kumar Tripathi[1,4,†], Sini Junttila[1,2,†], Tanja Buchacher[1,2,†], Rahul Biradar[1,2], Santosh D. Bhosale[1,5], Tapio Envall[1], Asta Laiho[1,2], Robert Moulder[1,2], Omid Rasool[1,2], Sanjeev Galande[3,6], Laura L. Elo[1,2,7,*] and Riitta Lahesmaa[1,2,7,*]

This file includes -

Materials and Methods

Supplementary figures and legends

Fig. S1. Expression profile of FOSL proteins in human Th17 cells

Fig. S2. Analysis of FOSL1 and FOSL2 levels in DKD and DOE Th17 cells

Fig. S3. Transcriptome analysis of FOSL DKD and DOE Th17 cells and experimental validation of their targets

Fig. S4. Validation of FOSL1 and FOSL2 co-regulated targets by immunoblot analysis

Fig. S5. Validation of FOSL1 and FOSL2 co-regulated targets by qRT-PCR analysis

Fig. S6. FOSL occupancy profile and their shared direct targets in human Th17 cells

Fig. S7. Genome-wide analysis of BATF gene targets in human Th17 cells

Fig. S8. Disease-linked SNPs at the shared binding sites of the three factors and overlapping with consensus AP-1 motifs

Fig. S9. DNA affinity precipitation assay (DAPA) of selected SNPs

Fig. S10. Densitometry analysis of DAPA immunoblots

METHODS

Western blotting

Cell culture pellets were lysed using RIPA buffer (Pierce, Cat. no. 89901), supplemented with protease and phosphatase inhibitors (Roche) and sonicated using Bioruptor UCD-200 (Diagenode, Seraing, Belgium). Sonicated lysates were centrifuged at 14,000 rpm for 20 min at 4°C and supernatants were collected. Samples were estimated for protein concentration (DC Protein Assay; Bio-Rad) and boiled with 6x Laemmli buffer (330 mM Tris-HCl, pH 6.8; 330 mM SDS; 6% β-ME; 170 μM bromophenol blue; 30% glycerol). Samples were loaded on gradient Mini-PROTEAN TGX Precast Protein Gels (BioRad, Helsinki, Finland) and transferred to PVDF membranes (Trans-Blot Turbo Transfer Packs, BioRad).

The following antibodies were used: anti-FOSL1 (Cell Signaling Tech, Cat. no. 5281), anti-FOSL2 (Cell Signaling Tech., Cat. no.19967); anti-STAT3 (Cell Signaling Tech., Cat. no. 9139); anti-BATF (Cell Signaling Tech., Cat. no. 8638), anti-STAT4 (Cell Signaling Tech., 2653); anti-NT5E/CD73 (Cell Signaling Tech., Cat. no. 13160); anti-APOD (Santa Cruz, Cat. no. sc-166612); anti-JUNB (Santa Cruz, Cat. no. sc-8051); anti-RORC (eBioscience, Cat. no. 14-6988-82) and anti-β-actin (SIGMA, Cat. no. A5441). HRP conjugated anti-mouse IgG (SantaCruz, Cat. no. sc-2005) and anti-rabbit IgG (BD Pharmingen, Cat. no. 554021) were used as secondary antibodies.

Flow cytometry

The following antibodies were used for flow cytometry: anti-CCR6 PE (BD Cat. no. 559562); anti-FOSL1 (Santacruz Biotechnology, Cat. no. sc-28310); anti-FOSL2 (Cell Signaling Tech., Cat. no.19967); anti-STAT3 (Cell Signaling Tech., Cat. no. 9139); APC-NT5E (CD73) monoclonal antibody (AD2) (Thermo Fischer, Cat. no.17-0739-42), PE anti-human CD70 antibody (Biolegend, Cat. no. 355103). For the primary antibodies that were unlabelled, the following secondary antibodies were used: Alexa 647 anti-mouse (Life Technologies, Cat. no. A21235) and Alexa 647 anti-rabbit (Life Technologies, Cat. no. A21245).

Anti-CCR6, anti-CD70 and anti-NT5E surface staining was performed either at 48h or 72h of Th17 polarization, as specified in the figure legends. Cells were washed twice with FACS buffer (0.5% FBS/ 0.1% Na-azide/PBS) and incubated with pre-labelled antibody for 20 min at 4ºC. For intracellular staining, cultured cells (24h or 72h) were fixed and permeabilized according to the manufacturer's instructions using IC staining buffers (Invitrogen, Cat nos. 00-5223-56; 00-5123-43; 00-8333-56). Cells were incubated with primary antibodies for 2h and subsequently washed using Perm Buffer. This was followed by 30-min incubations with labelled secondary antibodies. Suitable isotype or secondary antibody controls were maintained. Samples were acquired on LSRII (BD Biosciences, Franklin Lakes, NJ); live cells were gated based on forward and side scattering. The acquired data was analysed with FlowJo (FLOWJO, LLC).

Cytokine-induction assay

Naive CD4$^+$ T cells were cultured under the following conditions: CD3/CD28 activation (Th0), Th0 with IL-6, Th0 with IL-1β, Th0 with TGF-β, Th0 with IL-6 + IL-1β, Th0 with IL-6 + TGF-β, Th0 with IL-1β + TGF-β, and Th17 differentiation conditions (Th0 with IL-6 + IL-1β + TGF-β) for 24h. The neutralizing antibodies anti-IFN-γ and anti-IL-4 were added to each of these conditions. All cytokine and antibody concentrations were as described for Th17 culture conditions. FOSL1 or FOSL2 levels were estimated using Flow cytometry by performing Intracellular staining (described in the Flow Cytometry methods).

ELISA for IL-17 secretion

Secreted IL-17A levels were estimated using cell-culture supernatants of 72h cultured Th17 cells using either the Milliplex MAP human IL-17A kit (Merck Millipore; HCYTOMAG-60K-01), Bioplex human IL-17A Cytokine/chemokine 96-Well Plate Assay (Bio Rad; Cat. no. 171B5014M, 171304090M) or human IL-17A Duoset ELISA kit (R&D Biosystems DY317-05, DY008). The amount of IL-17A secreted by Th17 cells was normalized with the number of living cells determined based on forward and side scattering in flow cytometry analysis (LSRII flow cytometer; BD Biosciences).

Quantitative real-time PCR

Total RNA was isolated as described in '*RNA Isolation and RNA-Seq Sample Preparation' (Main methods section)*. cDNA was synthesized using SuperScript II Reverse Transcriptase and oligo(dT) primers as described in the manufacturer's instructions (Invitrogen, Cat nos. 18064-014 and 18418012). TaqMan primers and probes were designed with Universal Probe Library Assay Design Centre (Roche). All Taqman reactions were performed using Absolute QPCR Mix, ROX (Thermo scientific, Cat. no. AB1139A). EF1α was used as endogenous control. The qPCR runs were analysed using the 7900HT Fast Real-Time PCR System (Applied Biosystems). All Taqman primers and probes are listed in Table S1.

Ingenuity Pathway Analysis (IPA)

Pathway analysis was performed using Ingenuity Pathway Analysis (IPA, www.qiagen.com/ingenuity; Qiagen; March 2019) tool. IPA pathways with (Fisher's Exact Test corrected) p-value < 0.05 were considered as significantly enriched. Selected pathways involved in immune signaling are shown.

Immunofluorescence analysis

CD4$^+$ T cells were cultured for 72h under Th17 differentiation conditions and then spun down on poly-L-lysine-coated coverslips at 800 rpm. Cells were washed, fixed and permeabilized using Ebioscience Intracellular Staining kit (Invitrogen Cat nos.00-5223-56, 00-5123-43 and 00-8333-56). Permeabilized cells were further incubated overnight with primary antibodies against FOSL1 (Santacruz Biotechnology, Cat. no. sc-28310) or FOSL2 (Cell Signaling Tech, Cat. no.19967), and Lamin A/C (Santacruz Biotechnology, Cat. no. sc-7292). Cells were washed with Permeabilization buffer and further incubated for 60 mins with the respective anti-mouse or anti-rabbit Alexa flour secondary antibodies (Invitrogen Cat nos. A11031; A31572; A21202). Atto-Phalloidin A647 (Sigma, Cat. no. 65906) was used to stain cytoplasmic actin. Stained cells were finally mounted in Prolong Gold Antifade Mountant with DAPI (Life Technologies, Cat. no. P36941) and imaged on Zeiss 780 Confocal microscope.

Data representation for RNA-seq and ChIP-seq data

I. Heatmaps

a. Clustered heatmap for FOSL1, FOSL2 and BATF genomic binding: K-means clustered heatmap was generated using the 'PlotHeatmap' function from deepTools on Galaxy Europe, in order to visualize the genomic occupancy patterns of FOSL1, FOSL2 and BATF. Matrices of genomic coordinates were created using the ComputeMatrix tool, and a merged peak file of all three experiments (FOSL1, FOSL2 and BATF ChIP-seq) was used as a reference for generating the heatmap (1,2).

b. Heatmap for direct targets of FOSL1, FOSL2 and BATF: Common binding sites for FOSL1, FOSL2 and BATF obtained from ChIP-peak Anno analysis, were annotated to the nearest TSS using Homer. Of these, the genes oppositely regulated by FOSL and BATF in transcriptome analysis (FDR ≤ 0.1 and fold-change ≥ 1.5) were considered. Their corresponding RNA-seq expression changes were acquired and subsequent heatmaps were plotted using ‘plotHeatmap’ function from deepTools on Galaxy Europe (2).

II. Volcano plots for RNA-seq and ChIP-seq targets

a. Volcano plot for DKD, DOE and BATF KD RNA-seq targets: DE targets were acquired from RNA-seq analysis of DKD (24h and 72h), DOE (72h) or BATF KD (24h and 72h) Th17 cells. Volcano plots were generated using the ‘Volcano Plot’ function on Galaxy Europe (2). Targets with FDR ≤ 0.1 and fold change ≥ 1.8 are depicted as red (upregulated) and blue (downregulated) points. Selected Th17-relevant genes are labelled.

b. Volcano plot for FOSL1 and FOSL2 shared direct targets: FOSL1 and FOSL2 common binding sites obtained from ChIP-peak Anno analysis, were annotated to the nearest TSS using Homer. Of these, the genes that were differentially expressed in DKD or DOE (FDR ≤ 0.1 and fold-change ≥ 1.5) were considered. The corresponding RNA-seq expression changes for the listed targets were acquired and subsequent volcano plots were created as described above (2).

Cytoscape network for shared interactors of FOSL1 and FOSL2

The list of shared interactors for FOSL1 and FOSL2 was obtained from our recently published study (3). The common binding partners with known relevance to T-cell function were mapped against the STRING database. The assigned protein-protein interaction (PPI) network was further visualized using Cytoscape (4).

STRING interactome for BATF

A predictive interactome network for BATF was acquired using the STRING database (Only 'Text mining' and 'Experiments' were considered as the information source for the predicted partners). The minimum required interaction score was set to 0.7 (high confidence). The maximum number of interactors to be displayed in first shell was restricted to 10.

Immunoprecipitation

Immunoprecipitation of BATF was performed using Pierce MS-Compatible Magnetic IP Kit (Thermo Fischer, Cat. no.90409). 72h cultured Th17 cell pellets were lysed in appropriate volumes of cell-lysis buffer provided in the kit. BATF antibody (Cell Signaling Tech., Cat. no. 8638) or control rabbit IgG (Cell Signaling, Cat. no. 2729) was pre-incubated with protein A/G beads for 4–5 h to form antibody-bead complexes. Lysates were first pre-cleared with control IgG-bead complexes for 3 h. The pre-cleared lysates were then incubated overnight with BATF antibody-bead complexes (test IP) or control IgG-bead complexes (negative IP control). Immunoprecipitated protein complexes were washed (following manufacturer's protocol) and further eluted with appropriate volume of elution buffer. Eluted protein was run for immunoblotting.

Antibodies used for IP-immunoblotting are as follows: anti-BATF (Cell Signaling Tech, Cat. no. 8638); anti-RUNX1 A-2 (Santa Cruz Biotechnology, Cat. no. sc-365644); anti-JUNB C-11 (Santa Cruz Biotechnology, Cat. no.sc-8051); anti-STAT3 (Cell Signaling Tech., Cat. no. 9139); anti-IRF4 (P173) (Cell Signaling Tech., Cat. no. 4964); anti-SIRT1 (Cell Signaling Tech., Cat. no. 2496); anti-JUN (BD Biosciences, Cat. no.610326). Conformation-specific rabbit HRP (Cell Signaling Tech., Cat. no.5127) and mouse HRP (Cell Signaling Tech., Cat. no. 58802) were used as secondary antibodies.

SUPPLEMENTARY FIGURES AND LEGENDS

Fig. S1. Expression profile of FOSL proteins in human Th17 cells

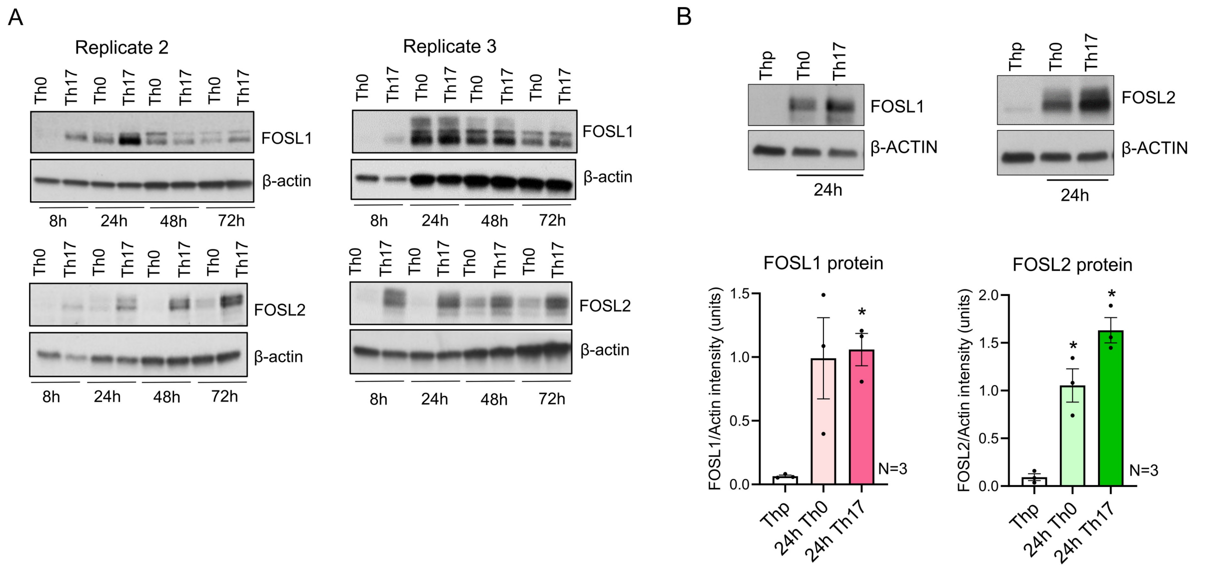

A Immunoblot images show FOSL1 (above) and FOSL2 (below) protein levels in naive CD4$^+$ T cells cultured under activation (Th0) or Th17 differentiation conditions, for the indicated time points. Actin has been used as loading control. Data represents biological replicates for Fig. **1B**. **B** Immunoblots depict FOSL1 (left) and FOSL2 (right) protein levels in naive CD4$^+$ T cells (Thp; 0 h time point) versus 24h cultured Th0/Th17 cells. Actin has been used as loading control. FOSL1 and FOSL2 levels (normalised to actin) are plotted in the graphs below. Data shows mean ± standard error of the mean (SEM) for three biological replicates. Statistical significance was calculated using two-tailed Student's t test (*p < 0.05).

Fig. S2. Analysis of FOSL1 and FOSL2 levels in DKD and DOE Th17 cells

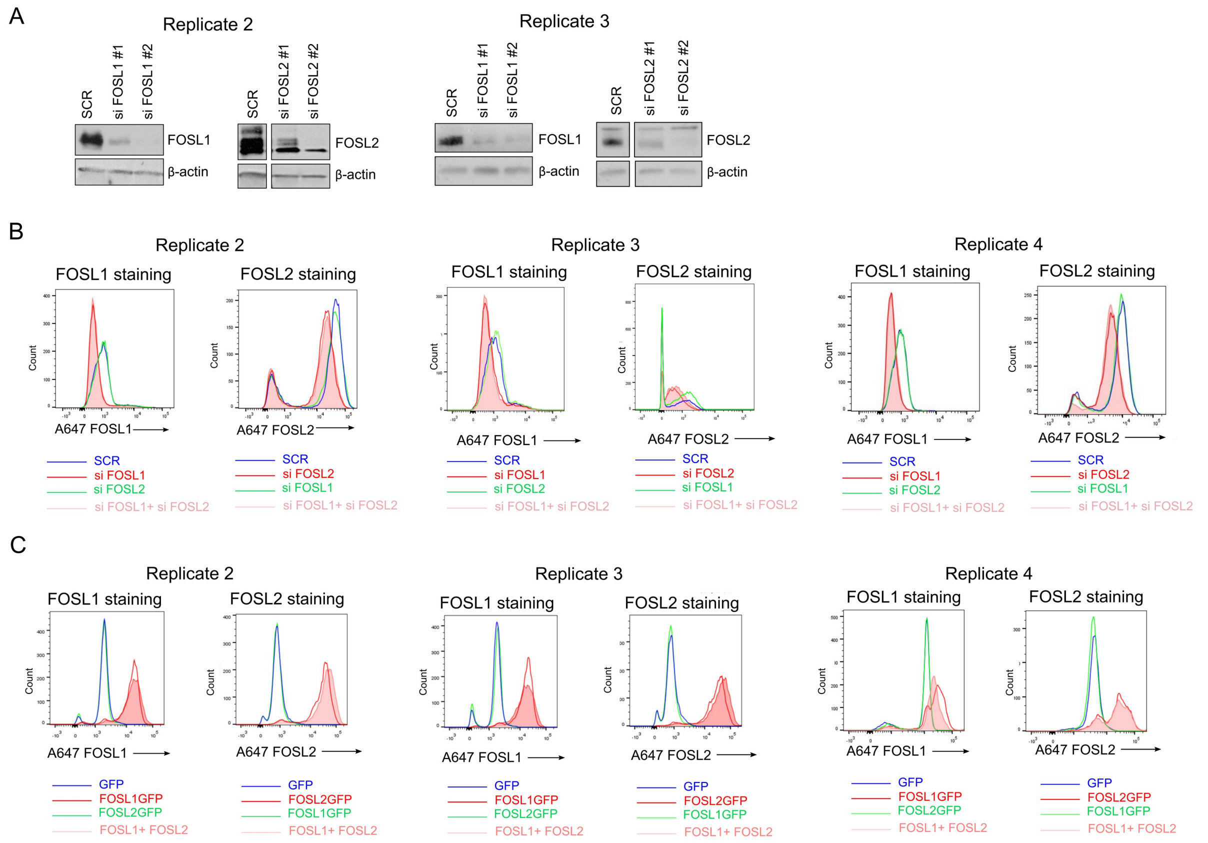

A Immunoblots depict FOSL1 and FOSL2 protein levels in naive CD4^{+} T cells that were silenced for the respective factors and further cultured under Th17-polarizing conditions for 24h. Non-targeting siRNA (Scramble or SCR) was used as nucleofection control and actin was used as loading control. Blots shown are biological replicates for Fig. **2A**. **B, C** FOSL KD/DKD (panel **B**) and FOSL OE/DOE (panel **C**) cells were labelled (Alexa-647) for total FOSL1 and FOSL2 protein. KD cells were labelled 24h post Th17-differentiation, whereas OE cells were labelled 18-20h (resting) post nucleofection. Expression of the corresponding factors was analysed using flow cytometry and overlay histograms were plotted (FOSL1, left; FOSL2, right). Panels B and C show biological replicates for Figs. **2C** and **2F**, respectively.

Fig. S3. Transcriptome analysis of FOSL DKD and DOE Th17 cells and experimental validation of their targets

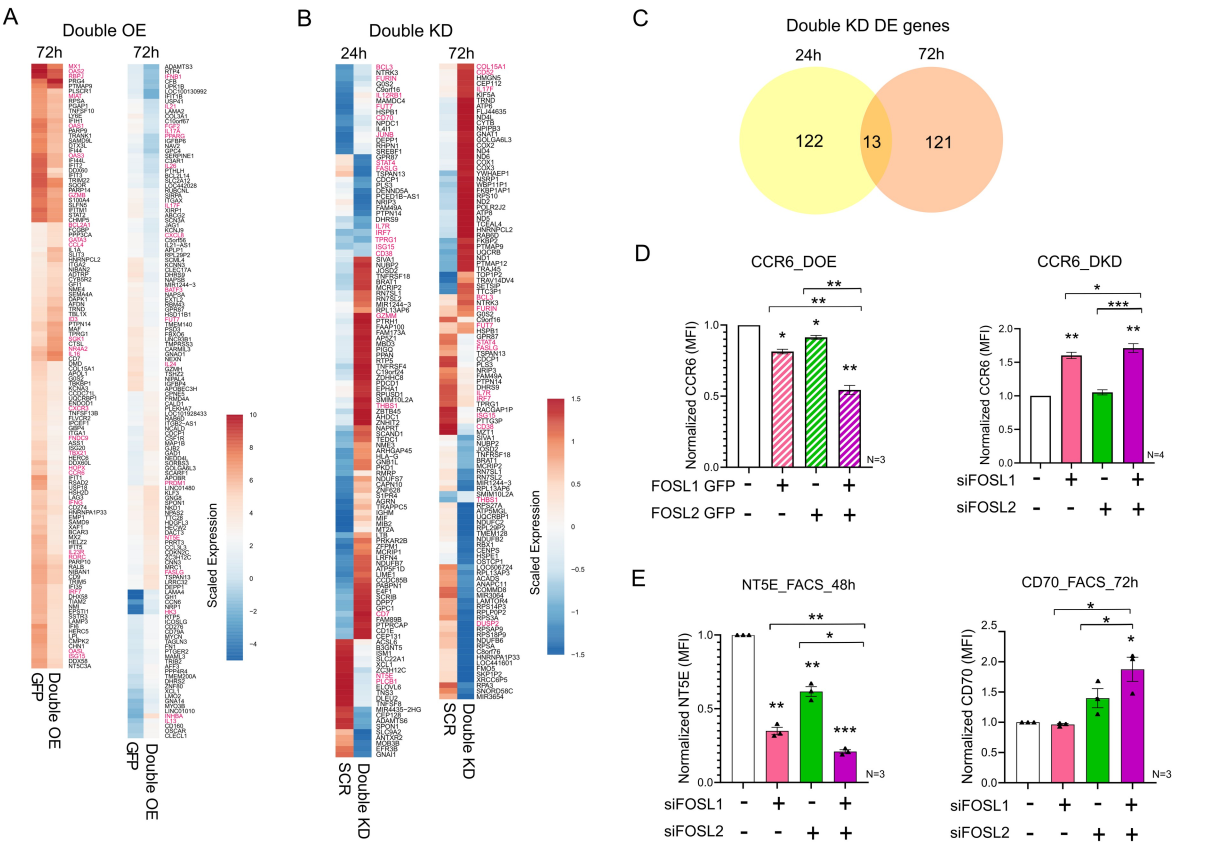

A, B Heatmap shows scaled expression values for the differentially expressed (DE) genes (FDR ≤ 0.1) in FOSL DOE (panel **A**) and FOSL DKD (panel **B**) Th17 cells. DOE targets with |fold change (FC)| ≥ 2 and DKD targets with |FC| ≥ 1.8 are selectively shown. For the DKD heatmap, only the genes with statistically significant expression values at both time points are depicted. Targets associated with Th17 or T helper cell differentiation are highlighted. **C** Venn diagram shows the overlap for the DKD gene-targets identified at 24h and 72h of Th17 polarization (FDR ≤ 0.1, |FC| ≥ 1.8). **D** CCR6 expression was analysed by flow-cytometry in FOSL OE/DOE (left) or KD/DKD (right) Th17 cells, at 72h of polarization. Median fluorescence intensity (MFI) values were normalized to the respective controls (SCR or Empty GFP) and plotted. **E** Figure shows flow-cytometry analysis of NT5E (left) and CD70 (right) protein expression in FOSL KD/DKD Th17 cells, at 48h and 72h of polarization, respectively. Bar plots show MFI values that are normalized to control. For panels **D** and **E**, data shows mean ± standard error of the mean (SEM) for three or four biological replicates, as indicated. Statistical significance was calculated using two-tailed Student's t test (*p < 0.05, **p < 0.01, ***p < 0.001).

Fig. S4. Validation of FOSL1 and FOSL2 co-regulated targets by immunoblot analysis

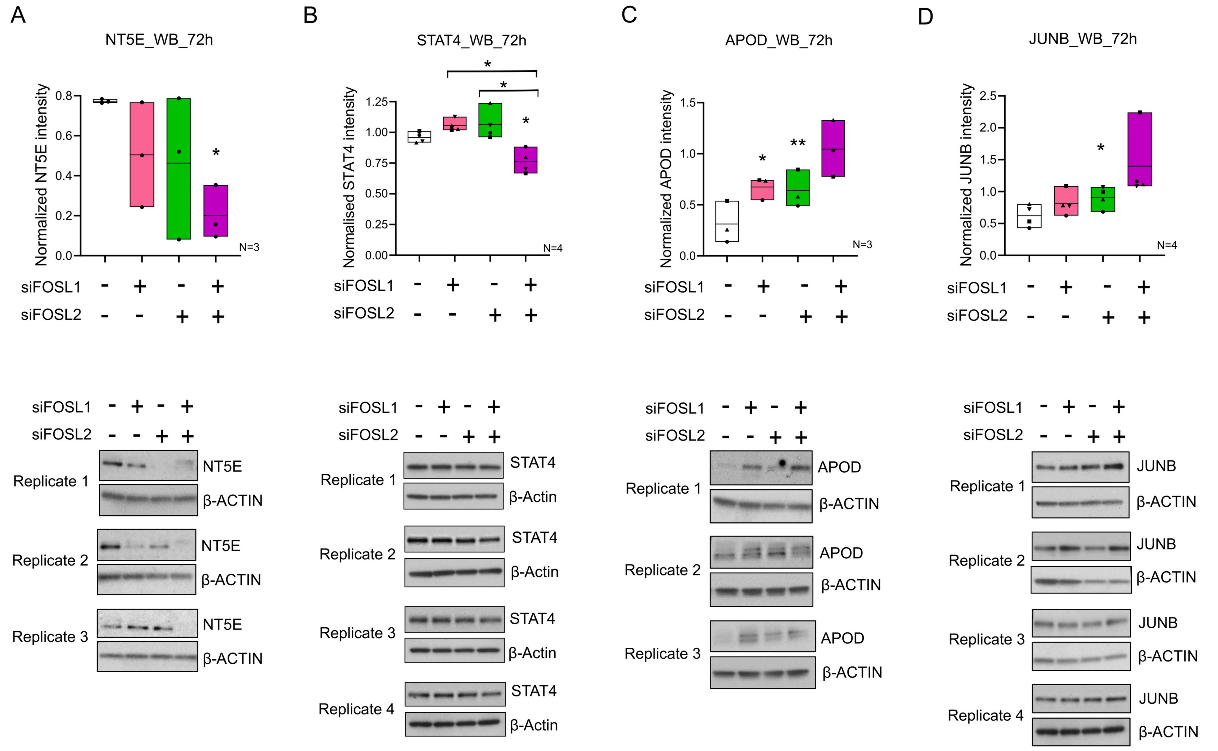

A-D Immunoblots (below) show protein-level expression of NT5E, STAT4, APOD and JUNB in FOSL KD and DKD Th17 cells at 72h of polarization. Actin serves as loading control. The JUNB and STAT4 blots for replicate 4 share the same loading control. Blots were quantified using ImageJ and the corresponding FOSL intensity values (normalised to actin) were plotted into graphs (above). Floating bars in the graphs indicate the minima to maxima values and the central line indicates the mean for three (NT5E, APOD) or four biological replicates (JUNB, STAT4). Statistical significance was calculated using two-tailed Student's t test (*$p < 0.05$; **$p < 0.01$).

Fig. S5. Validation of FOSL1 and FOSL2 co-regulated targets by qRT-PCR analysis

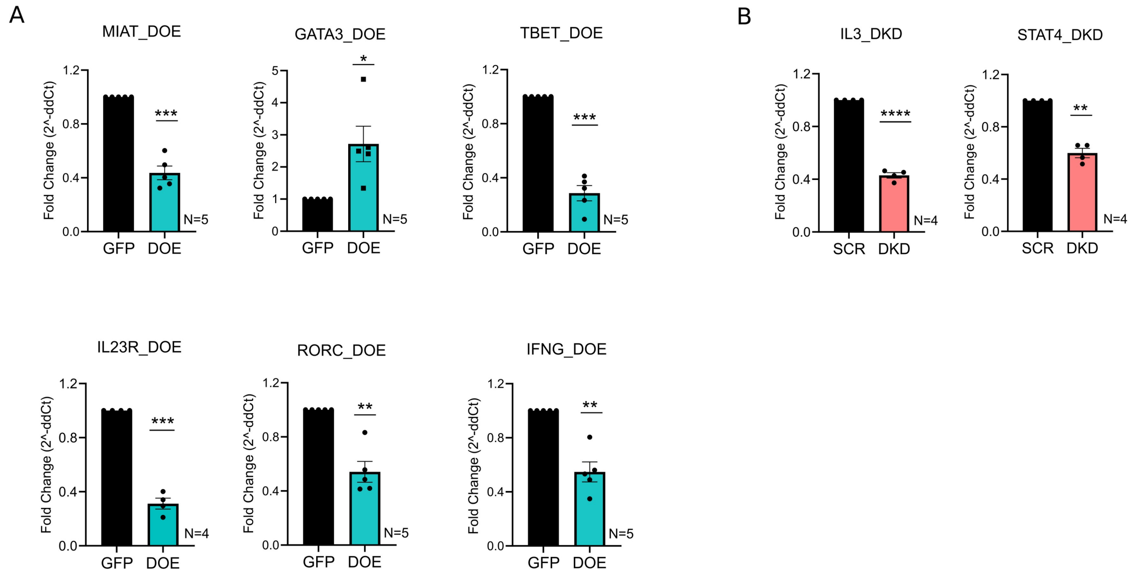

A, B qRT-PCR analysis was performed to validate the gene-expression changes in FOSL DOE Th17 cells at 72h of polarization (panel **A**), and in DKD Th17 cells at 24h of polarization (panel **B**). Fold change in expression with respect to control (SCR or GFP) is plotted. Data shows mean ± SEM for four or five biological replicates, as indicated. Statistical significance was calculated using two-tailed Student’s t test (*$p < 0.05$; **$p < 0.01$; ***$p < 0.001$; ****$p < 0.0001$).

Fig. S6. FOSL occupancy profile and their shared direct targets in human Th17 cells

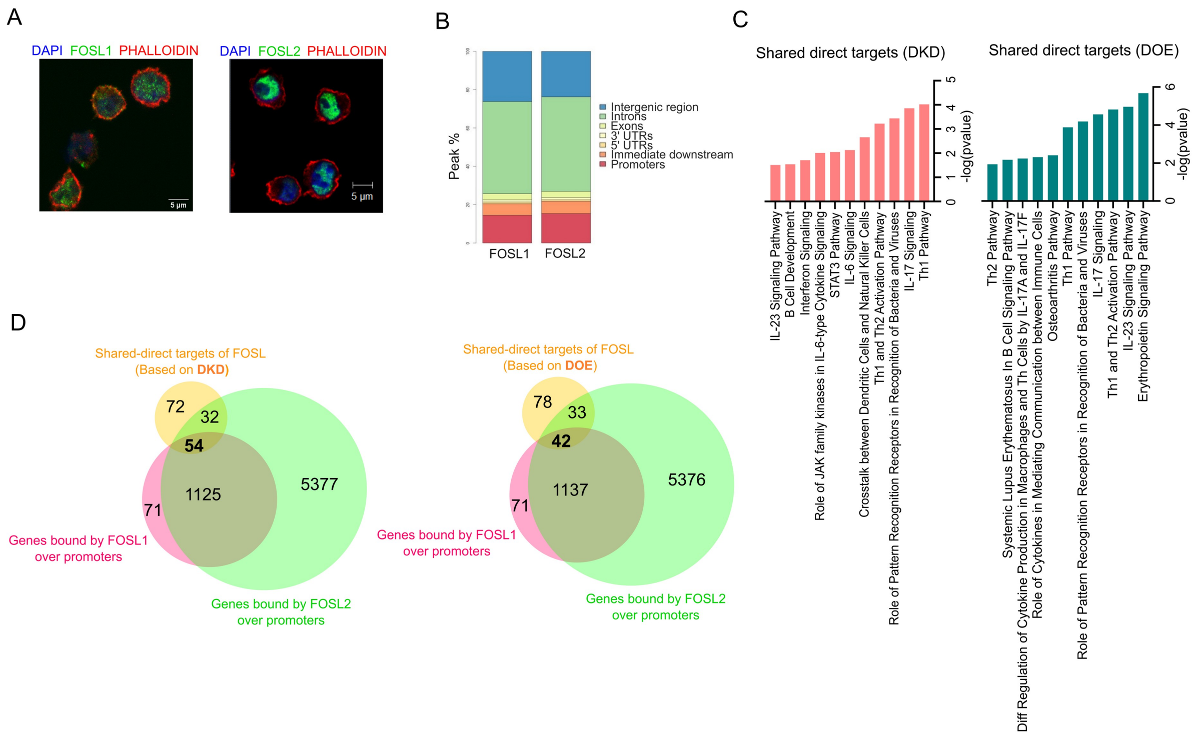

A Immunofluorescence analysis (replicate for Fig. **4A**) shows nuclear localization of FOSL1 (green, left panel) and FOSL2 (green, right panel) in 72h-polarized Th17 cells. Phalloidin (in red) stains the cytoplasmic actin and DAPI is shown in blue. **B** Bar plot depicts peak-annotation results for genomic binding sites of FOSL1 and FOSL2 in 72h Th17-polarized cells. **C** Genes that were co-regulated (i.e. DE under DKD or DOE conditions) and showed co-localized genomic-binding of FOSL1 and FOSL2, were annotated as their shared direct targets. These were enriched for signalling pathways using Ingenuity Pathway Analysis (IPA). The left and right panels show data for the DKD- and DOE-based shared direct targets, respectively. The top pathways related to T-cells and immune signaling are selectively shown. **D** Venn diagram highlights (in bold) the shared direct targets in DKD (left) and DOE (right) data, that are bound by FOSL factors over putative-promoter regions (5-kb around TSS). Of these, the Th17-associated hits are marked in the volcano plots of Fig. **4D**.

Fig. S7. Genome-wide analysis of BATF gene targets in human Th17 cells

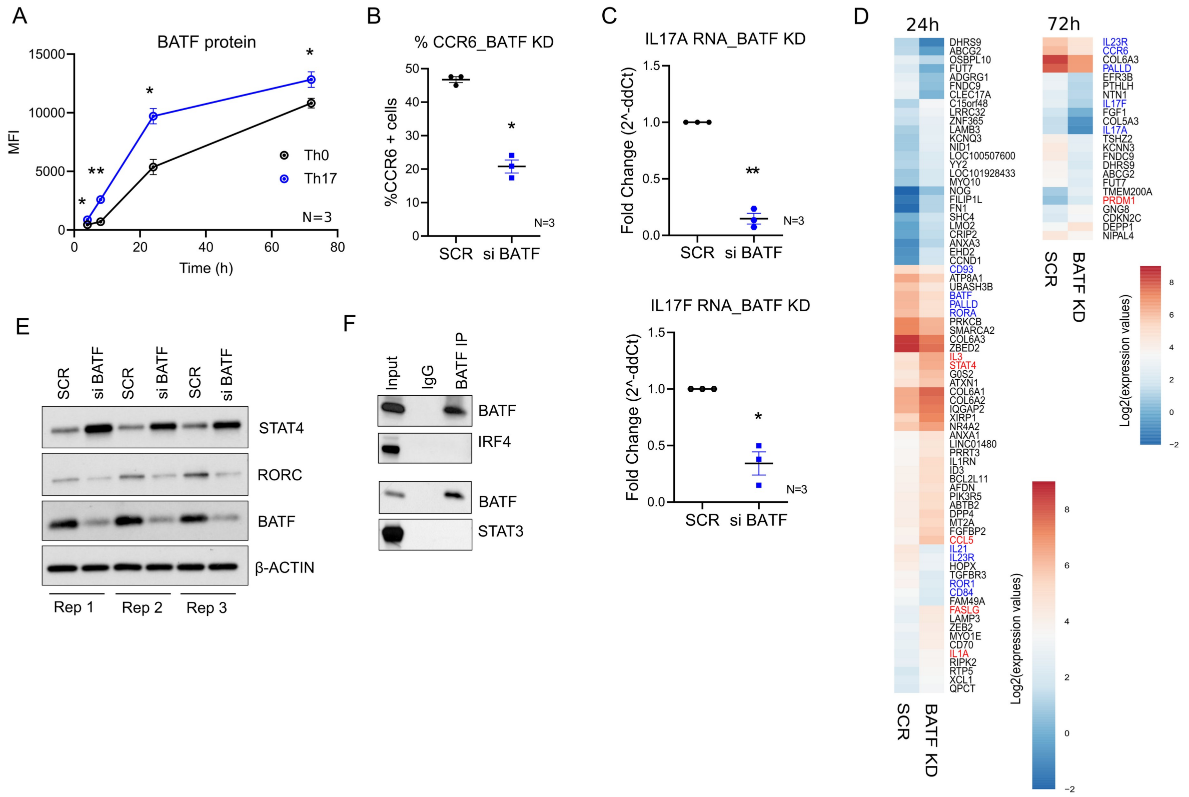

A Graph shows flow cytometry analysis of BATF protein levels in Th0 and Th17-polarizing cells, over a time-course. Data shows mean ± SEM for three biological replicates. Statistical significance was calculated using two-tailed Student's t test (*p < 0.05; **p < 0.01). **B**, **C** Panel **B** shows flow cytometry analysis of CCR6 positive cells (%) in SCR versus BATF-KD Th17 cells, at 72h of polarization. Panel **C** depicts qRT-PCR analysis of IL-17A (top) and IL-17F (bottom) RNA under the mentioned conditions. Data shows mean ± SEM for three biological replicates. Statistical significance was calculated using two-tailed Student's t test (*p < 0.05; **p < 0.01). **D** Heatmaps show the top DE genes (FDR ≤ 0.1, |FC| ≥ 1.8) in BATF-silenced Th17 cells at 24h (left) and 72h (right) of polarization. Scaled expression values are plotted and genes associated with Th17 cell-function are highlighted (upregulated genes are in red, downregulated ones are in blue). **E** Western blot analysis shows STAT4, RORC and BATF protein levels in SCR versus BATF KD Th17 cells, at 72h of polarization. Data for three biological replicates is shown and the quantified bar plot is provided as a part of Fig. **6F**. **F** BATF was immunoprecipitated from 72h-cultured Th17 cells and immunoblotting was performed to analyse its interaction with STAT3 and IRF4, which are reported to form pioneering complexes with BATF in murine Th17 cells.

Fig. S8. Disease-linked SNPs at the shared binding sites of the three factors and overlapping with consensus AP-1 motifs

rsID	Chr No	SNP Coordinates	Base Variant	Nearest Gene	SNP position at the motif (strand/nt)			AP-1 Motif Sequence Logo			Disease Association
					FOSL1	FOSL2	BATF	FOSL1	FOSL2	BATF	
rs17293632	15	67150258	C → T	RP11-342M21.2, SMAD3	+/7, -/4	+/7, -/4	+/8, -/3	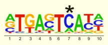	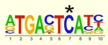	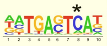	AS, CR,MS, PS,RA,UC
rs10808568	8	128251814	A → C	RN7SKP226, PVT1	+/1, -/10	+/1, -/10	+/2, -/9	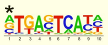	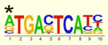	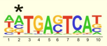	CD
rs6784841	3	69204481	G → A	LMOD3, FRMD4B	+/1, -/10	+/1, -/10	+/2, -/9	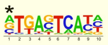	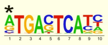	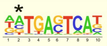	CD

Table shows information on the autoimmune-linked SNPs that are harboured within consensus AP-1 motifs at the shared genomic-binding sites of FOSL1, FOSL2 and BATF. The sequence logos shown have been derived from the respective TF ChIP-seq peaks using Homer.

Fig. S9. DNA affinity precipitation assay (DAPA) of selected SNPs

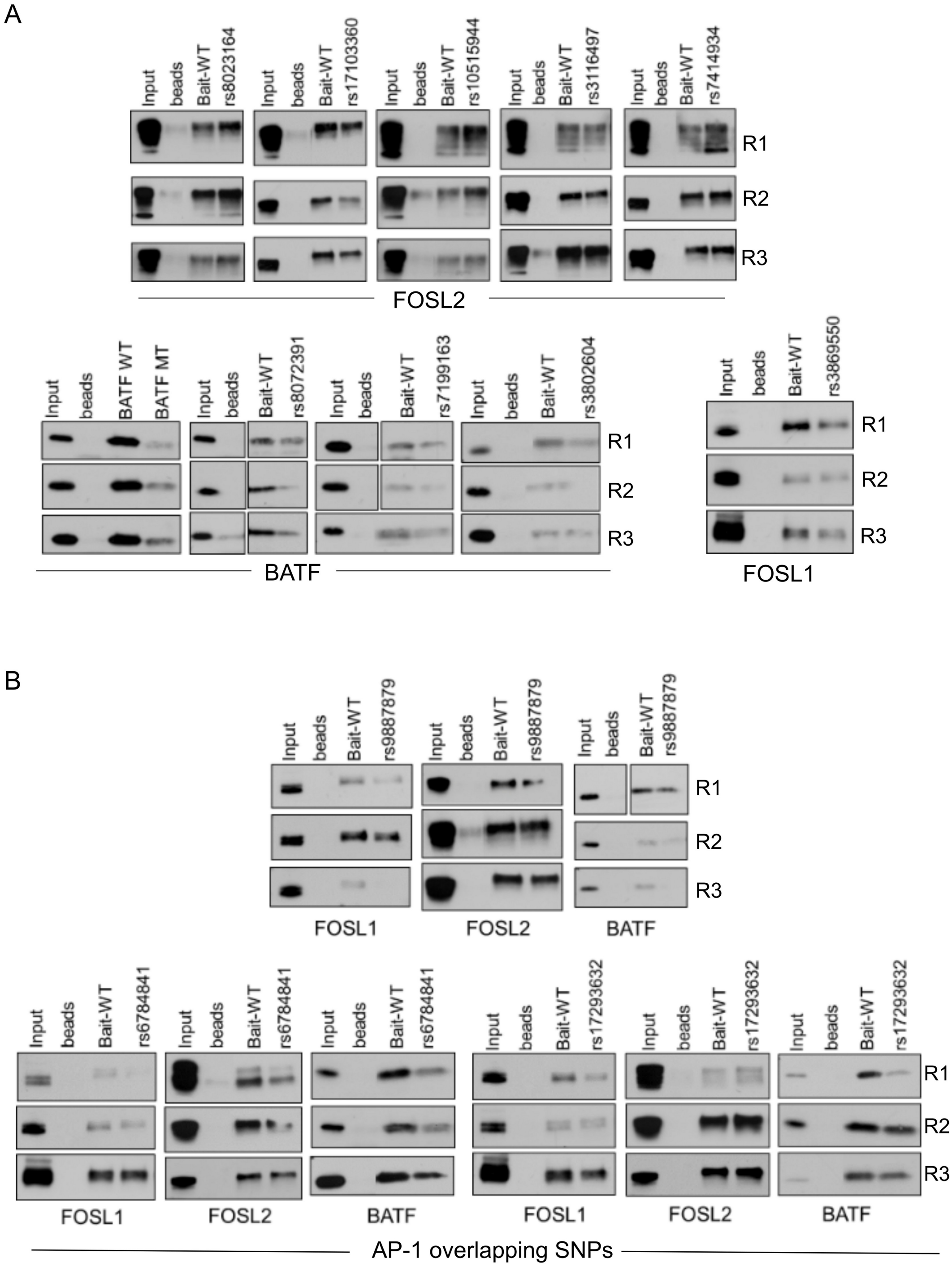

A, B DAPA analysis was performed to test the effect of selected SNPs on the DNA-binding abilities of FOSL1, FOSL2 and BATF. Panel **A** includes SNPs affecting the binding of either FOSL1, FOSL2 or BATF. Panel **B** depicts those SNPs at the common binding sites of the three factors which also alter the binding affinities for all of them. The common SNPs harboured within consensus AP-1 motifs are indicated. Immunoblots in panels **A** and **B** show biological replicates (R1, R2, R3) for Figs. **8C** and **D,** respectively.

Fig. S10. Densitometry analysis of DAPA immunoblots

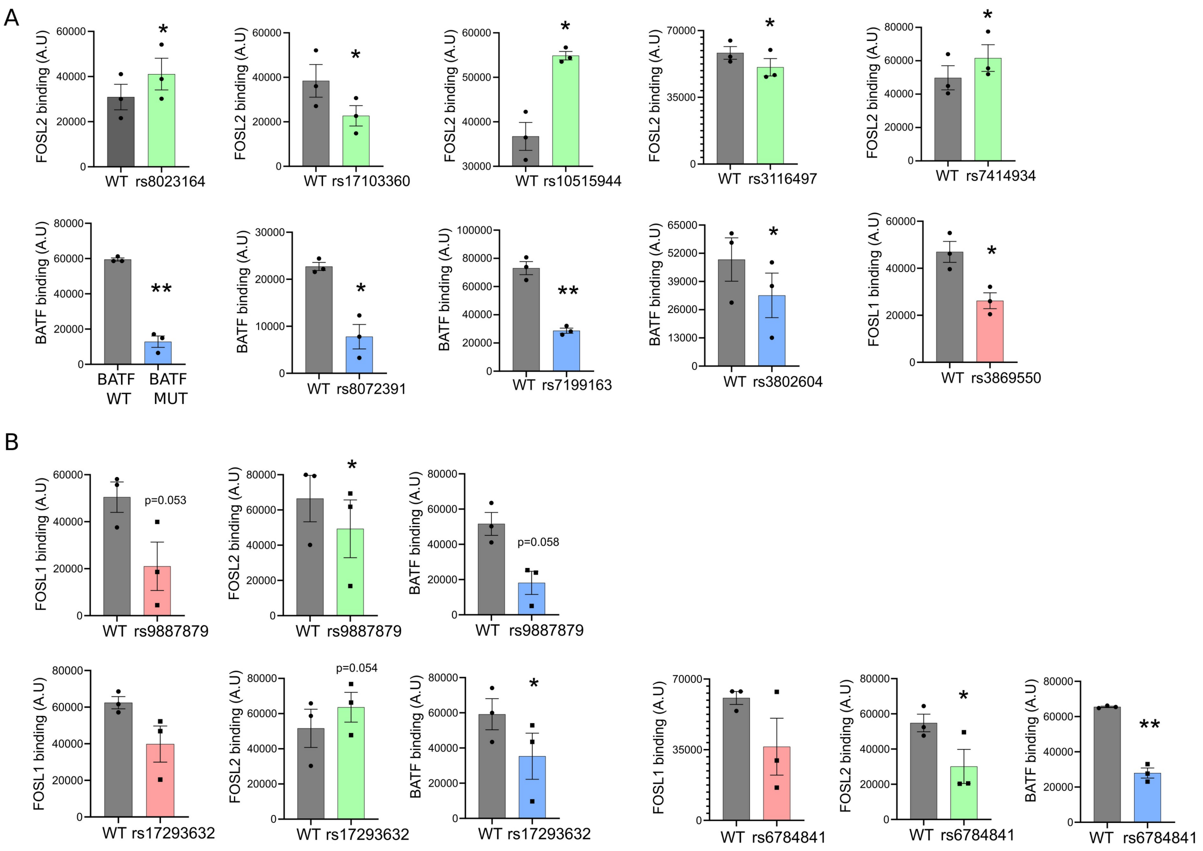

A, B DAPA immunoblots (Fig. **S9A, B**) were quantified using image J and the corresponding intensity values of FOSL1, FOSL2 and BATF were plotted for the WT versus MUT (containing the SNP) probe. Data shows mean ± SEM for three biological replicates. Statistical significance was calculated using two-tailed Student's t test (*p < 0.05; **p < 0.01).

REFERENCES

1. Ramírez, F., Ryan, D.P., Grüning, B., Bhardwaj, V., Kilpert, F., Richter, A.S., Heyne, S., Dündar, F. and Manke, T. (2016) deepTools2: a next generation web server for deep-sequencing data analysis. *Nucleic acids research*, **44**, W160-W165.
2. Afgan, E., Baker, D., Van den Beek, M., Blankenberg, D., Bouvier, D., Čech, M., Chilton, J., Clements, D., Coraor, N. and Eberhard, C. (2016) The Galaxy platform for accessible, reproducible and collaborative biomedical analyses: 2016 update. *Nucleic acids research*, **44**, W3-W10.
3. Shetty, A., Bhosale, S.D., Tripathi, S.K., Buchacher, T., Biradar, R., Rasool, O., Moulder, R., Galande, S. and Lahesmaa, R. (2021) Interactome Networks of FOSL1 and FOSL2 in Human Th17 Cells. *ACS omega*, **6**, 24834-24847.
4. Shannon, P., Markiel, A., Ozier, O., Baliga, N.S., Wang, J.T., Ramage, D., Amin, N., Schwikowski, B. and Ideker, T. (2003) Cytoscape: a software environment for integrated models of biomolecular interaction networks. *Genome research*, **13**, 2498-2504.
