# Supplementary material for: A systematic comparison of FOSL1, FOSL2 and BATF-mediated transcriptional regulation during early human Th17 differentiation
Source: Nucleic Acids Res. 2022 May 3;50(9):4938–58. doi: 10.1093/nar/gkac256 (PMC9122603; doi:10.1093/nar/gkac256)
Supplement: gkac256_Supplemental_Files [file gkac256_supplemental_files.zip › Revised Captions and Legends for Supp Excel sheets_Shetty et al (002).docx]

**Legends and Captions for Supplementary Files**

**Table S1. siRNA and Taqman primer sequences**

List of siRNA sequences for RNAi experiments and primer and probe sequences for qRT-PCR analysis.

**Table S2. DE genes_FOSL1, FOSL2, Double KD**

List of differentially expressed (DE) genes detected by RNA-seq in FOSL1 KD, FOSL2 KD and double KD (DKD) Th17 cells.

**Table S3. DE genes_FOSL1, FOSL2, Double OE**

List of DE genes detected by RNA-seq in FOSL1 OE, FOSL2 OE and double OE (DOE) Th17 cells.

**Table S4. FOSL1 FOSL2 ChIP, shared direct targets, motif analysis**

List of individual and shared genomic binding sites of FOSL1 and FOSL2 detected by ChIP-seq. In addition, their shared direct targets and known TF-motifs within their binding sites are listed.

**Table S5. BATF DE genes, ChIP peaks, direct targets and motif analysis**

RNA-seq data showing BATF-regulated genes and ChIP-seq data showing BATF-bound genomic sites in Th17 cells. In addition, genes directly regulated by BATF and known TF-motifs within its binding sites are listed.

**Table S6. BATF ChIP peaks shared with FOSL**List of genomic binding sites shared between FOSL1, FOSL2 and BATF.

**Table S7. Disease-linked SNPs within TFBS & Common SNPs**List of SNPs linked to autoimmune diseases that are harbored within the individual or shared (common) genomic binding sites of FOSL1, FOSL2 and BATF.

**Table S8. List of SNPs relevant to the study**
List of disease-linked SNPs that overlap with FOSL1, FOSL2 and BATF binding sites in the vicinity of Th17-relevant genes. In addition, SNPs at the shared binding sites of these factors that are harbored within consensus AP-1 motifs are listed.

**Table S9. Oligo sequences for DAPA**List of oligonucleotides used for DAPA.

**Table S10. Overlap with HuTh17 codeset and STAT3 data**

Overlap of FOSL gene-targets with HuTh17 codeset genes (*Hu et al., 2017 Nat. Commun.*) and STAT3-regulated genes (*Tripathi et al., 2017 Cell Reports*)
